# MFGE‐8, a Corona Protein on Extracellular Vesicles, Mediates Self‐Renewal and Survival of Human Pluripotent Stem Cells

**DOI:** 10.1002/jev2.70056

**Published:** 2025-03-25

**Authors:** Youngseok Lee, Hyojin Kim, Heeseok Yoon, Seunghyun Cho, Jeongjun Kim, Jihun Lee, Sang‐Hun Choi, Hyesun Cho, Dong‐Hun Woo, Jung‐Hyuck Park, Choongseong Han, Jong‐Hoon Kim

**Affiliations:** ^1^ Laboratory of Stem Cells and Tissue Regeneration, Department of Biotechnology, College of Life Sciences and Biotechnology Korea University Seoul South Korea; ^2^ Institute of Animal Molecular Biotechnology Korea University Seoul South Korea; ^3^ Department of Neuroscience Yale School of Medicine New Haven Connecticut USA; ^4^ Department of New Drug Development NEXEL Co., Ltd. Seoul South Korea

**Keywords:** extracellular vesicles, human pluripotent stem cells, MFGE‐8, protein corona, stemness

## Abstract

Extracellular vesicles (EVs) and secretory factors play crucial roles in intercellular communication, but the molecular mechanisms and dynamics governing their interplay in human pluripotent stem cells (hPSCs) are poorly understood. Here, we demonstrate that hPSC‐secreted milk fat globule‐EGF factor 8 (MFGE‐8) is the principal corona protein at the periphery of EVs, playing an essential role in controlling hPSC stemness. MFGE‐8 depletion reduced EV‐mediated self‐renewal and survival in hPSC cultures. MFGE‐8 in the EV corona bound to integrin α_v_β_5_ expressed in the peripheral zone of hPSC colonies. It activated cyclin D1 and dynamin‐1 via the AKT/GSK3β axis, promoting the growth of hPSCs and facilitating the endocytosis of EVs. Internalization of EVs alleviated oxidative stress and cell death by transporting redox and stress response proteins that increased GSH levels. Our findings demonstrate the critical role of the extracellular association of MFGE‐8 and EVs in modulating the self‐renewal and survival of hPSCs.

## 1 Introduction

Autocrine and paracrine signalling, which are critical in embryonic development, have emerged as key regulators of the stemness and differentiation of pluripotent stem cells (PSCs) (Ho et al. 2015; Hur et al. 2021). However, the molecular mechanisms of cellular communication between human PSCs (hPSCs) and their impact on stemness regulation are largely unknown. Major players in cell–cell communication include secretory factors (SFs) and extracellular vesicles (EVs) (Daneshmandi et al. 2020; van Niel et al. 2022). Numerous SFs accumulate around the periphery of EVs through molecular interactions, forming a stable structure called the protein corona. This corona layer determines the extracellular functions of EVs, facilitating the bridging of EVs to the surfaces of recipient cells (Buzas 2022; Santucci et al. 2019; Choi et al. 2021). Therefore, the peripheral proteins surrounding EVs, initially considered contaminants of EV preparation, were recently recognized as critical regulators of EV‐mediated intercellular communication (Buzas 2022; Tóth et al. 2021; Wolf et al. 2022; Gomes et al. 2022; Lee and Kim 2022). Previous studies suggested that the secretory peptide ELABELA from human embryonic stem cells (hESCs) (Ho et al. 2015) and EVs from mouse ESCs (Hur et al. 2021) may serve as autocrine regulators of self‐renewal and pluripotency. However, the synergistic interactions between SFs and EVs are poorly understood and the protein corona around hPSC‐derived EVs (hPSC‐EVs) has not been reported to date.

EVs contain different spectra of proteins and nucleic acids depending on their cellular origin (Daneshmandi et al. 2020; van Niel et al. 2022; Buzas 2022; Jeppesen et al. 2023). Upon internalization, EVs release their luminal cargo into the cytoplasm of recipient cells, altering the intracellular environment and cellular behaviour. Therefore, EVs from young donors and tissue‐resident stem cells may mitigate age‐related damage in adult tissues (Yoshida et al. 2019; Fafián‐Labora et al. 2020; Zhang et al. 2019; Lou et al. 2017). MicroRNAs in EVs have drawn increasing interest as important cargoes capable of altering gene expression in recipient cells (Ying et al. 2017; Zhang et al. 2017). More recently, enzymes delivered by EVs have been shown to counteract aging and disease progression, suggesting a direct influence of protein cargoes on various metabolic processes (Yoshida et al. 2019; Fafián‐Labora et al. 2020). While it has been observed that hPSC‐EVs exhibit potentials to rejuvenate or regenerate adult cells and tissues (Zhang et al. 2019; Xia et al. 2021; Hu et al. 2020; Povero et al. 2019; Kmiotek‐Wasylewska et al. 2024), the mechanisms and specific cargoes responsible for these effects are largely unknown.

Under defined culture conditions, hPSCs are capable of self‐renewal on Matrigel in the absence of other cell types, such as mouse embryonic fibroblast feeder cells (MEFs). However, there may be subtle differences in the properties of individual hPSCs depending on their position in the colony. Within an hPSC colony, the environment at the edges differs from that of the interior zone (Rosowski et al. 2015), but nearly all cells express the pluripotency transcription factors (TFs). Thus, there is likely a mechanism for shuttling signals between hPSCs to harmonize and maintain stemness. In this study, we integrated cell biology, biochemistry and proteomics to characterize the roles of SFs and EVs in self‐renewal and survival of hPSCs. We found that a glycoprotein MFGE‐8 is released from hPSCs and plays a crucial role in EV‐mediated intercellular communications. Furthermore, we delineated the dynamics and molecular pathways underlying the interactions between MFGE‐8 and EVs and identified protein cargoes in hPSC‐EVs associated with the self‐renewal and survival of hPSCs.

## 2 Materials and Methods

### 2.1 Cell Culture and Reagents

The hESC line BG01 (WiCell Research Institute) and the hiPSC line HDF01‐hiPSCs (derived from human dermal fibroblasts, generously supplied by Dr. Yong‐Mahn Han, KAIST, Korea), were cultured on plates coated with Matrigel (Corning) in mTeSR1 medium (Stemcell Technologies) supplemented with 1% (v/v) penicillin‐streptomycin (Gibco), which was replaced every 24 h. For the initial seeding, hPSCs were placed in mTeSR1 medium supplemented with 10 µM ROCK inhibitor Y‐27632 (Tocris Bioscience). Human BJ fibroblasts (American Type Culture Collection) were maintained in Dulbecco's Modified Eagle Medium (DMEM, Gibco) supplemented with 10% (v/v) foetal bovine serum (Gibco), 1% (v/v) penicillin‐streptomycin, 1× MEM non‐essential amino acids solution (Gibco) and 0.1 mM β‐mercaptoethanol (Sigma‐Aldrich). For co‐culture of hESCs with human BJ fibroblasts, hESCs were plated at a density of 1 × 10^4^ cells/well in Matrigel‐coated 24‐well tissue culture plates (Corning) containing 500 µL of mTeSR1 medium. One hour after the initial seeding of hESCs, 3 × 10^4^ human BJ fibroblasts were added to the cultured hESCs. The mixed culture was then incubated in mTeSR1 medium containing 5 µg/mL mouse IgG (Sigma‐Aldrich) or an antibody that neutralizes MFGE‐8 activity (anti‐M8nAb, MFG‐06 [sc‐8029], Santa Cruz Biotechnology). The culture medium and anti‐M8nAb were refreshed every 24 h throughout the experiment. Samples were collected every 24 h over a period of 4 days. Human bone marrow‐derived mesenchymal stem cells (BM‐hMSCs, Lonza) were maintained using the MSCGM Mesenchymal Stem Cell Growth Medium BulletKit (Lonza). All cells were cultured in a humidified incubator at 37°C with a 5% CO_2_ atmosphere. The cell counts and growth curves for each cell type were assessed using the LUNA‐II (Logos Biosystems) automated cell counter. CHIR99021 (Tocris Bioscience), LY294002 (Thermo Fisher), RGE peptide (GRGESP, AnaSpec), RGD peptide (GRGDSP, Sigma‐Aldrich), mouse anti‐integrin α_v_β_5_ (R&D Systems), *N*‐ethyl‐*N*‐isopropyl amiloride (EIPA, Sigma‐Aldrich), Dynasore (Sigma‐Aldrich), filipin III (Santa Cruz Biotechnology), chlorpromazine (Sigma‐Aldrich) and buthionine sulfoximine (BSO, Sigma‐Aldrich) were administered directly to hESC or HDF01‐hiPSC cultures at the specified concentrations and durations to modulate the activities of signalling components, endocytic machinery molecules or glutathione (GSH) metabolism.

### 2.2 Immunofluorescence Staining

Cells were fixed in 4% (v/v) paraformaldehyde (Sigma‐Aldrich) for 20 min at room temperature (RT), followed by three washes with sterile phosphate‐buffered saline (PBS). Fixed cells were blocked with 10% (v/v) donkey serum (Abcam) in sterile PBS, with or without 0.1% (v/v) Triton X‐100 (Sigma‐Aldrich), for 45 min at RT. Cells were then incubated overnight with primary antibodies at 4°C, washed in sterile PBS and incubated with the secondary antibody for 90 min at RT in the dark. The cells were washed three times with sterile PBS, and the cell nuclei were stained with 1.0 µg/mL of DAPI (4′,6‐diamidino‐2‐phenylindole dihydrochloride, Sigma‐Aldrich) for 10 min at RT. Fluorescent images were captured using a confocal laser scanning microscope (LSM800, Carl Zeiss), and ImageJ (NIH) software was used to determine the percentage of immunoreactive cells. The following primary antibodies were used: mouse anti‐OCT4 (1:400; sc‐5279, Santa Cruz Biotechnology), rabbit anti‐vimentin (1:200; 5741, Cell Signalling Technology), goat anti‐E‐cadherin (1:400; AF648, R&D Systems), mouse anti‐integrin α_v_β_5_ (1:100; MAB2528, R&D Systems), mouse anti‐8‐oxoguanine (1:200; MAB3560, Millipore) and rabbit anti‐53BP1 (1:200; 4937, Cell Signalling Technology). The following secondary antibodies were used: donkey anti‐mouse IgG Alexa Fluor 488 (1:400; A21202, Invitrogen), donkey anti‐mouse IgG Alexa Fluor 568 (1:400; A10037, Invitrogen), donkey anti‐mouse IgG Alexa Fluor 647 (1:400; A31571, Invitrogen), donkey anti‐rabbit IgG Alexa Fluor 568 (1:400; A10042, Invitrogen) and donkey anti‐goat IgG Alexa Fluor 488 (1:400; A11055, Invitrogen).

### 2.3 5‐ethynyl‐2′‐deoxyuridine (EdU) incorporation assay

The EdU assay was conducted using the Click‐iT EdU Cell Proliferation Kit (C10337, Invitrogen). BG01‐hESCs and HDF01‐hiPSCs were exposed to 10 µM EdU for 15 min, and human BJ fibroblasts and BM‐hMSCs for 24 h. After two washes in sterile PBS, cells were fixed with 4% (v/v) paraformaldehyde for 20 min and permeabilized with 0.5% (v/v) Triton X‐100 in sterile PBS containing 10% (v/v) donkey serum for 45 min. After three washes in sterile PBS, cells were immunostained as described above. EdU incorporation was determined according to the manufacturer's protocol. Fluorescent images were captured using a confocal laser scanning microscope (LSM800, Carl Zeiss). EdU‐positive cells were quantified using ImageJ software.

### 2.4 Hoechst 33342 and Propidium Iodide Staining

Cell death was measured by Hoechst 33342 (Thermo Fisher) and propidium iodide (PI) staining solution (PI, BD Biosciences). Briefly, BG01‐hESCs and HDF01‐hiPSCs were cultured in DMEM/F‐12 medium (Gibco) supplemented with 20% (v/v) KnockOut serum replacement (Gibco), 1% (v/v) penicillin‐streptomycin, 1× MEM non‐essential amino acids solution and 0.1 mM β‐mercaptoethanol, with or without extracellular vesicles (EVs), antibodies or other indicated reagents. After treatment, cells were incubated with sterile PBS containing Hoechst 33342 and PI for 10 min at 37°C and observed by fluorescence microscopy (Apotome‐Axiovert 200 M, Carl Zeiss). PI‐positive cells were quantified using ImageJ software.

### 2.5 Spontaneous Differentiation of Human Pluripotent Stem Cells

Spontaneous differentiation of hESCs was induced on Matrigel using only DMEM/F‐12 medium supplemented with 1% (v/v) penicillin‐streptomycin, 1× MEM non‐essential amino acids solution and 0.1 mM β‐mercaptoethanol, without KnockOut serum replacement or fibroblast growth factor 2 (FGF2). Briefly, hESCs were cultivated to confluence on Matrigel‐coated 60‐mm tissue culture dishes (Corning), gently dissociated with ReLeSR (Stemcell Technologies), and then approximately 8% of the total dissociated cell aggregate was delicately replated as a large clump in each well of Matrigel‐coated 12‐well tissue culture plates (Corning) containing 1 mL of mTeSR1 medium. After 24 h, the culture medium was replaced with 1 mL of DMEM/F‐12, and the medium was changed every other day. On the day specified in each experiment, cells were sampled for analysis.

### 2.6 Reverse Transcription‐Quantitative PCR

Total RNA was isolated from the cells using the TRIzol reagent (Invitrogen), and cDNA was synthesized using the RevertAid Reverse Transcription (RT) Kit (Thermo Fisher). RT‐quantitative polymerase chain reaction (RT‐qPCR) assays using iQ SYBR Green SuperMix (Bio‐Rad) were performed in triplicate using the CFX‐96 Real‐Time PCR Detection System (Bio‐Rad). The primer sequences are provided in the Table S1. The relative expression levels of the target genes were determined after normalizing expression to the mRNA levels of housekeeping genes, glyceraldehyde 3‐phosphate dehydrogenase (GAPDH) or beta‐actin (ACTB), using the 2^−DDCt^ method.

### 2.7 Preparation of Conditioned Medium, Soluble Secretory Factors (SFs) and EVs

**FIGURE 1:**
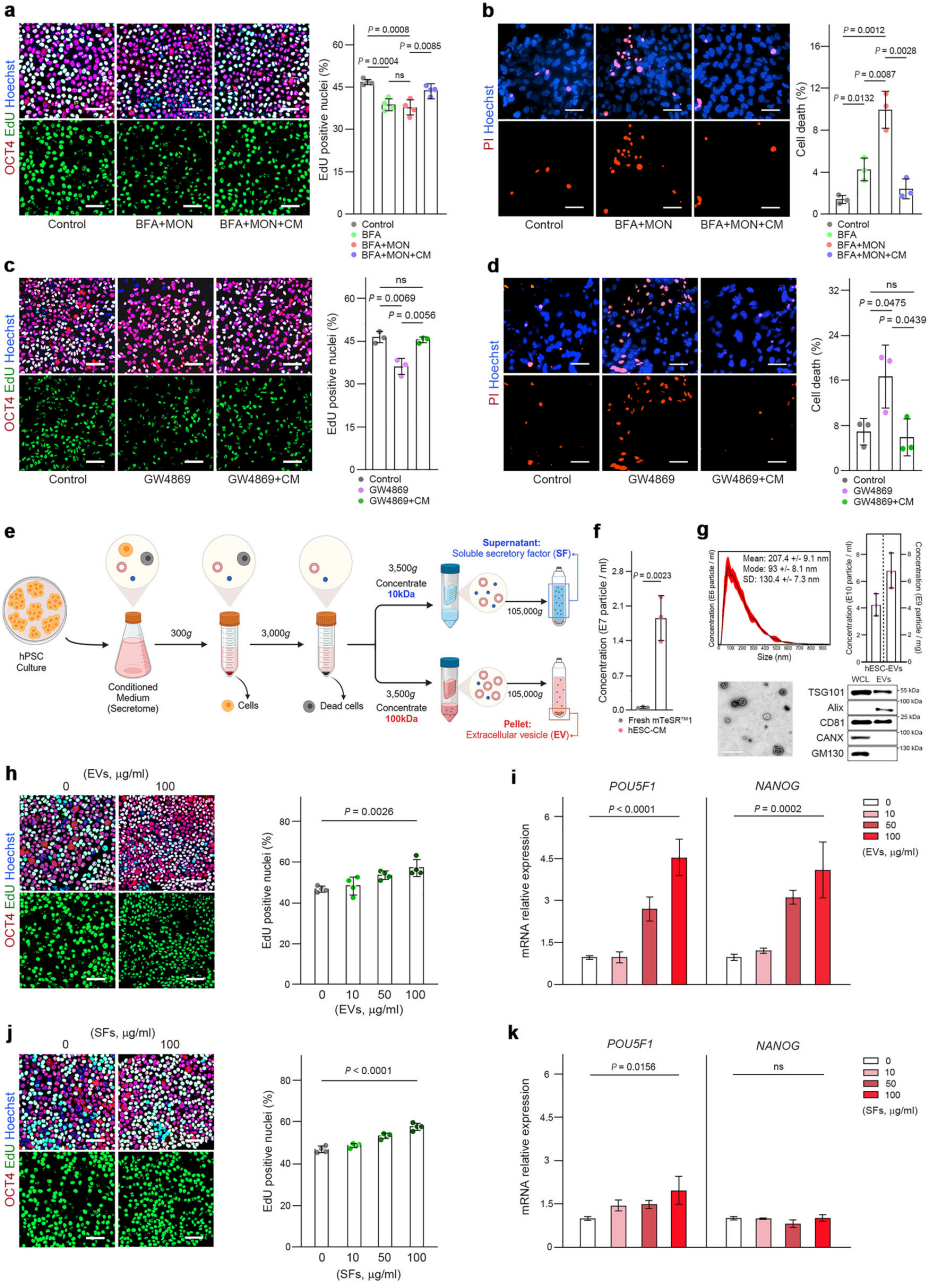
Endogenous EVs and SFs secreted by hESCs contribute to the maintenance of stemness. (a,c) hESCs labelled with EdU 6 h after treatment with PBS (control), protein secretion inhibitor BFA, either alone or in combination with MON (BFA + MON) (a), or GW4869 (c) with or without the secretome from conditioned medium (CM) of hESCs (a, *n* = 4; c, *n* = 3). Cells were stained for OCT4 and counterstained with Hoechst 33342. (b,d) hESCs were treated with PBS, BFA, BFA + MON (b) or GW4869 (d) with or without CM for 12 h, and cell death was evaluated by propidium iodide (PI) staining (*n* = 3). Nuclei were stained with Hoechst 33342. (e) The procedure for separating EVs and SFs in CM from hESC cultures. (f) Particle numbers in fresh mTeSR1 medium and the medium conditioned by hESCs (hESC‐CM). hESCs were grown at 70%−80% confluency for 24 h in mTeSR1. The particle numbers were measured by nanoparticle tracking analysis (NTA) (*n* = 3). (g) Characterization of hESC‐EVs: top left, NTA histogram showing sizes and concentrations of EVs; top right, particle concentrations of the isolated hESC‐EV samples. The particle concentration in EV solutions was determined by NTA and expressed in particle/mL and particle/mg; lower left, transmission electron micrograph of EVs; lower right, immunoblots of TSG101, Alix, CD81, CANX and GM130 in whole cell lysates (WCL) and EVs. (h,j) hESCs were treated with increasing amounts of EVs (h) or SFs (j) for 2 days, and proliferation was measured by EdU incorporation (*n* = 4). Cells were stained for OCT4, and nuclei were stained with Hoechst 33342. (i,k) RT‐qPCR analysis of *POU5F1* and *NANOG* expression in hESCs that were differentiated spontaneously for 5 days with increasing doses of EVs (i) or SFs (k) (*n* = 3). The graph values are mean ± S.D., with *p* values from two‐tailed *t*‐tests (a–d,f) or one‐way ANOVA (h–k). Scale bars (a–d,h,j), 50 µm. Scale bar (g), 1 µm.

EVs were isolated from conditioned medium (CM) by differential centrifugation as described previously (Adnani et al. 2022) (Figure 1e). Briefly, CM was harvested from BG01‐hESCs or HDF01‐hiPSCs cultured in mTeSR1 medium for 24 h at 70%−80% confluency. Cells and cell debris were removed by centrifugation at 300 × *g* for 5 min, followed by 3000 *× g* for 15 min. The supernatant was then processed differently depending on whether EV or SF to be isolated. For SFs, the supernatant was concentrated by centrifugation at 3500 *× g* for 20 min in an Amicon Ultra‐15 Centrifugal Filter Unit with Ultracel 10,000 Dalton MWCO filters (Millipore). The concentrate was then subjected to an additional centrifugation at 105,000 *× g* for 2 h at 4°C in an Optima XE‐90 Ultracentrifuge (Beckman Coulter) using a SW 41 Ti Swinging‐Bucket Rotor (Beckman Coulter). The resulting supernatant was used as SFs for further experiments. To purify EVs, we concentrated the supernatant by centrifugation at 3500 *× g* for 20 min in an Amicon Ultra‐15 Centrifugal Filter Unit with Ultracel 100,000 Dalton MWCO filters (Millipore). EVs were then pelleted from the concentrate by centrifugation at 105,000 *× g* for 2 h at 4°C in an Optima XE‐90 Ultracentrifuge (Beckman Coulter) using a SW 41 Ti Swinging‐Bucket Rotor (Beckman Coulter). The pelleted EVs were washed with sterile EV‐free PBS and centrifuged again at 105,000 × *g* for 2 h at 4°C. The resulting EV pellet was then resuspended in EV‐free PBS or RIPA buffer for subsequent experiments.

### 2.8 Nanoparticle Tracking Analysis

The size distribution and number of particles in fresh mTeSR1 medium, CM and purified EVs obtained from an hPSC culture were determined by nanoparticle tracking analysis (NTA) with the NanoSight LM10 instrument (Malvern Instruments Ltd.). To compare the particle numbers between fresh mTeSR1 medium and hESC‐CM, complete mTeSR1 medium was used, and CM was obtained from hESC cultures following the same procedure described above. To measure the size distribution and particle number of purified EVs, 20 µg samples (based on protein content) were diluted in 400 µL EV‐free PBS. To measure the changes in EV diameter by generating the protein corona, 20 µg of bare (proteinase K‐treated) EVs were incubated with EV‐free PBS or 18‐fold concentrated hESC‐SFs in 100 µL EV‐free PBS at 37°C for 24 h. The samples were then diluted to a final volume of 400 µL with EV‐free PBS. The Brownian motion was recorded for 60 s and analysed with NTA v.2.3 software (Malvern Panalytical) using the Stokes−Einstein equation to calculate the hydrodynamic diameter.

### 2.9 Western Blot

EVs and hPSCs were lysed in 1× RIPA buffer (Biosolution) containing PhosSTOP and cOmplete protease inhibitor cocktails (Roche) and centrifuged at 13,000 *× g* for 20 min at 4°C. The protein concentrations in the supernatant were determined using the BCA Protein Assay Kit (Sigma‐Aldrich), following the manufacturer's instructions. Proteins (10 or 20 µg) from cell lysates, EVs and SFs were separated by sodium dodecyl sulphate‐polyacrylamide gel electrophoresis (SDS‐PAGE) in NuPAGE 1.0–1.5 mm, 4%–12% Bis‐Tris mini protein gels (Invitrogen). The proteins were transferred onto Immun‐Blot PVDF membrane (polyvinylidene difluoride, Bio‐Rad), incubated with Ponceau S solution (Thermo Fisher) at RT for 30 s and washed three times in TBST (Tris‐buffered saline [Calbiochem] containing 0.1% [v/v] Tween 20 [Sigma‐Aldrich]) to remove background staining. The membrane was blocked in 5% (w/v) Blotting Grade Blocker non‐fat dry milk (Bio‐Rad) or bovine serum albumin (BSA, Bovogen) in TBST for 1 h at RT. The membrane was incubated with the primary antibody overnight at 4°C and then with the secondary antibody conjugated with horseradish peroxidase (HRP) for 1 h at RT. The primary antibodies included: rabbit anti‐TSG101 (1:4000; ab125011, Abcam), mouse anti‐Alix (1:500; 2171, Cell Signalling Technology), mouse anti‐CD81 (1:1000; sc‐166029, Santa Cruz Biotechnology), mouse anti‐GSTP1 (1:1000; 3369, Cell Signalling Technology), rabbit anti‐calnexin (1:2000; ab22595, Abcam), mouse anti‐GM130 (1:1000; 610822, BD Biosciences), mouse anti‐HSP90α/β (1:1000; sc‐13119, Santa Cruz Biotechnology), mouse anti‐OCT4 (1:2000; sc‐5279, Santa Cruz Biotechnology), rabbit anti‐Nanog (1:2000; 4903, Cell Signalling Technology), rabbit anti‐GAPDH (1:4000; sc‐25778, Santa Cruz Biotechnology), mouse anti‐MFGE‐8 (1:2000; 278901 [MAB2767], R&D Systems), rabbit anti‐β‐actin (1:1000; 4970, Cell Signalling Technology), rabbit anti‐phospho‐Ser473 Akt (1:5000; 4060, Cell Signalling Technology), rabbit anti‐phospho‐Ser9 GSK3β (1:4000; 5558, Cell Signalling Technology), rabbit anti‐Akt (1:2000; 9272, Cell Signalling Technology), rabbit anti‐GSK3β (1:2000; 610201, BD Biosciences), rabbit anti‐dynamin‐1 (1:2000; ab52852, Abcam), rabbit anti‐phospho‐Ser774 dynamin‐1 (1:2000; ab55324, Abcam), rabbit anti‐cyclin D1 (1:2000; 2978, Cell Signalling Technology) and rabbit anti‐phospho‐Thr286 cyclin D1 (1:2000; ab62151, Abcam). The secondary antibodies included goat anti‐mouse IgG HRP (1:10,000−100,000; 31430, Invitrogen) and donkey anti‐rabbit IgG HRP (1:10,000−100,000; ab6802, Abcam). The immunoblots were visualized using WestGlow ECL chemiluminescent substrate (Biomax). Images were acquired and analysed using ImageQuant LAS4000 (GE Healthcare) with the ImageQuant LAS 4000 Control Software (GE Healthcare). The intensity of the protein bands on the western blots was quantified using ImageJ software, and the values were normalized to GAPDH (e.g., pS^473^Akt/GAPDH) or the total amount of the targeted protein (e.g., pS^473^Akt/Akt).

### 2.10 Blocking the Release of Secretory Proteins or Extracellular Vesicles

hESCs (4 × 10^4^ cells) seeded in individual wells of Matrigel‐coated 24‐well tissue culture plates containing 500 µL of mTeSR1 medium were grown for 3 days and then treated with: (1) 1× eBioscience Brefeldin A Solution (containing 1000× brefeldin A, Invitrogen), (2) 1× eBioscience Protein Transport Inhibitor Cocktail (containing 500× brefeldin A and monensin, Invitrogen) to inhibit protein secretion, (3) 2 µg/mL GW4869 (Sigma‐Aldrich) to inhibit exosome secretion or (4) 50% (v/v) CM to supplement both secretory proteins and exosomes. These treatments were carried out at 37°C for 6, 12 or 24 h, depending on the experiment.

### 2.11 Proteinase K Treatment

Bare EVs were generated by proteinase K (Sigma‐Aldrich) treatment of intact hESC‐EVs as previously described (van de Vlekkert et al. 2019). Briefly, EVs were harvested from hESC culture and treated with 100 µg/mL proteinase K for 3 h at 37°C. The EVs were then incubated with 5 mM phenylmethylsulfonylfluoride (PMSF) for 10 min to deactivate the proteinase K, and bare EVs were harvested by centrifugation at 105,000 × *g* for 2 h at 4°C and resuspended in sterile EV‐free PBS or RIPA buffer.

### 2.12 Zeta Potential Measurement

The zeta potential of hESC‐EVs was determined by phase analysis light scattering using a NanoBrook 90Plus instrument (Brookhaven Instruments). To identify a corona on the surface of EVs, 40 µg of EVs or bare EVs were suspended in 50 µL of sterile EV‐free PBS or 18‐fold concentrated SFs, incubated at 37°C for 24 h, and diluted with 1.5 mL of EV‐free PBS. Zeta potential was measured using a solvent‐resistant electrode (Brookhaven Instruments) inserted into a BI‐SCGO 4.5 mL cuvette (Brookhaven Instruments) containing the sample.

### 2.13 Transmission Electron Microscopy

To analyse EV morphology, each sample was resuspended in sterile distilled water and subjected to negative staining transmission electron microscopy. A 10 µL drop of the sample was placed on Formvar‐carbon‐coated copper grids (Electron Microscopy Sciences) for 15 s, then gently removed using filter paper. A 1% (w/v) uranyl acetate solution (Electron Microscopy Sciences) was applied for 30 s to negatively stain the sample, followed by removal with filter paper. The grid was rinsed with a drop of sterile distilled water. The samples were then examined using the HT7800 TEM (HITACHI) at the Yonsei Biomedical Research Institute, Yonsei University College of Medicine.

### 2.14 Pre‐Treatments of Extracellular Vesicles With SFs, Reagents or Antibodies

Intact or bare EVs were exposed to SFs, recombinant human MFGE‐8 protein (rhMFGE‐8, R&D Systems), recombinant human MFGE‐8 ΔC1 (NEXEL Co., Ltd), recombinant human MFGE‐8 ΔC2 (NEXEL Co., Ltd.) or three anti‐MFGE‐8 neutralizing antibodies (MFG‐06 [sc‐8029, Santa Cruz Biotechnology]; F‐5 [sc‐271574, Santa Cruz Biotechnology]; 278901 [MAB2767, R&D Systems]) at indicated concentrations and for a designated time at 37°C. This pre‐treatment was carried out before administering the EVs to cell cultures.

### 2.15 Live Imaging

The hESC line BG01 was treated with 5 µg/mL anti‐M8nAb (MFG‐06 [sc‐8029, Santa Cruz Biotechnology]) or 5 µg/mL mouse IgG for 48 h. The bright field live cell images were taken using JuLI Br (NanoEntek).

### 2.16 RNA Sequencing and Analysis

The hESC line BG01 was treated with 5 µg/mL anti‐M8nAb (MFG‐06 [sc‐8029, Santa Cruz Biotechnology]) or 5 µg/mL mouse IgG for 12 h or 24 h. The total RNA was isolated from cells using TRIzol reagent and the RNA sequencing data were obtained from the Beijing Genomics Institute (GEO: GSE276921). Raw reads were trimmed using Trimmomatic (Bolger et al. 2014), and subsequently aligned to the human genome (GRCh38.p14) according to the STAR 2‐pass method (Dobin et al. 2013). Duplicated reads were eliminated using Picard Markduplicate (http://broadinstitute.github.io/picard). According to Ensembl gene set, the trimmed reads were aligned to the genes using HTSeq (Anders et al. 2015). Fragments per kilobase of transcript per million mapped reads (FPKM) for each gene was calculated by RNAnorm and used for gene set enrichment analysis (GSEA). GSEA (version 4.3.3) was conducted with the gene sets from Molecular Signatures Database (MSigDB) (Mootha et al. 2003; Subramanian et al. 2005). To normalize read counts for differential gene expression profiling, we utilized the variance stabilizing transformation method of DESeq2. The *p* values were adjusted to *q* values for multiple testing by Benjamini‐Hochberg correction. Genes exhibiting two‐fold change in mean expression with *q* value < 0.05 in anti‐M8nAb‐treated cells compared to isotype IgG‐treated cells at each time point (12 and 24 h) were identified as differentially expressed genes (DEGs). Principal component analysis (PCA) was visualized using the plotPCA function in DESeq2. Hierarchical clustering analysis of DEGs was performed and visualized as heatmaps using the pheatmap package. The Venn diagram of common upregulated and downregulated DEGs was generated by jvenn (Bardou et al. 2014). Gene ontology enrichment analysis of common DEGs were performed using DAVID (*p* value < 0.05) (Huang da et al. 2009; Sherman et al. 2022). Bubble plots of GSEA and GO enrichment analysis results were created using SRplot (Tang et al. 2023). The functional interaction network of common upregulated DEGs were analysed by ClueGO plug‐in in Cytoscape (version 3.9.1) using gene ontology (GO): biological process terms (Shannon et al. 2003; Bindea et al. 2009).

### 2.17 Protein Digestion for Proteomic Analysis

The lysed EVs in 1X RIPA buffer containing the PhosSTOP (Roche) and cOmplete Protease Inhibitor Cocktail (Roche) were resuspended in 8 M urea using an Amicon Ultra Centrifugal 0.5 mL‐3K filter. The protein concentration was quantified using the Pierce BCA Protein Assay Kit (Thermo Fisher), and digestion was performed using filter‐aided sample preparation in a Microcon 30K centrifugal filter device (Millipore). Samples were reduced by incubating them with Tris(2‐carboxyethyl)phosphine at 37°C for 30 min and alkylated with iodoacetic acid at 25°C for 1 h in the dark. After sequential washing with lysis buffer and 50 mM ammonium bicarbonate, the proteins were digested with trypsin at an enzyme‐to‐protein ratio of 1:50 (w/w) at 37°C for 18 h. The peptide mixtures were moved to new tubes, and trypsin was inactivated by acidifying with 15 µL of formic acid (Honeywell). The digested peptides were desalted using C18 spin columns (Harvard Apparatus) and eluted with 80% acetonitrile in 0.1% formic acid in water.

### 2.18 LC‐MS/MS Analysis

The samples in 0.1% formic acid in water were analysed using a Q‐Exactive Orbitrap hybrid mass spectrometer (Thermo Fisher) and an Ultimate 3000 system (Thermo Fisher). We used a 2 cm × 75 µm ID trap column packed with 3 µm C18 resin and a 50 cm × 75 µm ID analytical column packed with 2 µm C18 resin, depending on the hydrophobicity of the peptides. The mobile phase solvents were: (A) 0.1% formic acid in water and (B) 0.1% formic acid in 80% acetonitrile, and the flow rate was 300 nL/min. The gradient of the mobile phase was as follows: 4% solvent B for 14 min, 4%–15% solvent B for 61 min, 15%–28% solvent B for 50 min, 28%–40% solvent B for 20 min, 40%–96% solvent B for 2 min, holding at 96% solvent B for 13 min, 96%–4% solvent B for 1 min and 4% solvent B for 24 min. A data‐dependent acquisition method was adopted, and the top ten precursor peaks were selected and isolated for fragmentation. Ions were scanned at high resolution (70,000 in MS1, 17,500 in MS2 at *m*/*z* 400), and the MS scan range was 400–2000 *m*/*z* at both the MS1 and MS2 levels. Precursor ions were fragmented with a normalized collisional energy (NCE) value of 27%. Dynamic exclusion was set to 30 s.

### 2.19 Proteome Data Analysis

Thermo MS/MS raw files for each analysis were searched using Proteome Discoverer software (ver. 2.5) with the human proteome database from UniProt. The consensus workflow included a peptide‐spectrum match validation step and used SEQUEST HT as a database search algorithm. The search parameters were 20 ppm of tolerance of precursor ion masses, 0.02 Da fragment ion mass, and a maximum of two missed cleavages with trypsin enzyme. The dynamic modifications on peptides included static carbamidomethylation of cysteine (+57.012 Da), variable modifications of methionine oxidation (+15.995 Da), N‐terminal acetylation of protein (+42.011 Da), and N‐terminal carbamylation of protein (+43.0006 Da). The search results below 1% of false discovery rate (FDR) were selected and filtered for peptides of at least six amino acids in length. Data analysis and graphic visualization were performed using web‐based platforms Enrichr (https://maayanlab.cloud/Enrichr/ for transcription factor enrichment analysis) (Chen et al. 2013; Kuleshov et al. 2016; Xie et al. 2021), DAVID (https://david.ncifcrf.gov for gene ontology analysis) (Huang da et al. 2009; Sherman et al. 2022), KOBAS‐i (http://39.103.204.200/ for KEGG and Reactome pathway analysis) (Bu et al. 2021), and Metascape (https://metascape.org) (Zhou et al. 2019). Possible protein–protein interactions (PPIs) were inferred using GeneMANIA in Cytoscape (Shannon et al. 2003; Warde‐Farley et al. 2010), which also predicts potential proteins associated with the target proteins.

### 2.20 Enzyme‐Linked Immunosorbent Assay (ELISA)

Human MFGE‐8 in samples was quantified using the Human MFGE‐8 Quantikine ELISA Kit (R&D Systems) according to the manufacturer's instructions.

### 2.21 Alkaline Phosphatase Cell Staining

hESCs (8 × 10^4^ cells) were plated in wells of Matrigel‐coated 12‐well tissue culture plates that contained 1 mL of mTeSR1 medium supplemented with either 10 µg/mL rhMFGE‐8 or an equivalent volume of PBS without the ROCK inhibitor Y‐27632. After 24 h, the culture medium was replaced with 1 mL of mTeSR1 medium and refreshed daily. On day 7, the cells were fixed in a 4% (v/v) paraformaldehyde solution for 20 min at RT, washed three times in sterile PBS, and incubated with the Vector Blue AP Substrate Kit (Vector Laboratories) according to the manufacturer's protocol. The alkaline phosphatase‐positive cells, stained blue, were quantified using ImageJ software.

### 2.22 EV Uptake Analysis

Intact or bare hESC‐EVs were purified by ultracentrifugation and labelled with a lipophilic fluorescent dye using the PKH26 Red Fluorescent Cell Linker Kit (Sigma‐Aldrich) according to the manufacturer's instructions. For EV uptake experiments, 4 × 10^4^ hPSCs were seeded in each well of a Matrigel‐coated 24‐well tissue culture plate containing 500 µL of mTeSR1 medium. Three days after cell seeding, the cells were exposed to PKH26‐labelled EVs (PKH‐EVs) at concentrations of 10 or 30 µg/mL for the time period indicated. As indicated, hPSCs or EVs were treated with SFs, reagents or antibodies prior to the administration of EVs. After incubation with PKH‐EVs, cells were washed three times with sterile EV‐free PBS before immunofluorescence staining. Fluorescent images were captured using a confocal laser scanning microscope (LSM800, Carl Zeiss). To quantify EV uptake by recipient hPSCs, we measured the fluorescence intensity of PKH26 accumulation in colonies using ImageJ software.

### 2.23 Flow Cytometry

To measure the binding of hESC‐EVs to integrins on the surface of hESCs, we plated cells at a density of 6 × 10^4^ cells per well in Matrigel‐coated 12‐well tissue culture plates containing 1 mL of mTeSR1 medium. After 3 days, the cells were exposed to 30 µg/mL of EVs for 6 h, then harvested using TrypLE Select Enzyme (Gibco), and incubated with 100 µM RGD‐FITC (GRGDSP, AnaSpec) for 30 min at 4°C in sterile EV‐free PBS containing 3% (v/v) BSA. After three washes, cells were resuspended in 100 µL of EV‐free PBS containing 3% (v/v) BSA for flow cytometry to quantify RGD‐FITC bound to the hESCs. To evaluate cell cycle progression, we seeded hESCs at a density of 5 × 10^4^ cells per well in Matrigel‐coated 12‐well tissue culture plates containing 1 mL of mTeSR1 medium. After 2 days, the cells were treated with 5 µg/mL of mouse anti‐M8nAb (MFG‐06 [sc‐8029], Santa Cruz Biotechnology) or 5 µg/mL of mouse IgG for 48 h, harvested using TrypLE Select Enzyme, and fixed in 70% (v/v) ethanol at 4°C overnight. The cell cycle of fixed hESCs was assessed by flow cytometry using the BD Accuri C6 Plus Flow Cytometer (BD Biosciences) after staining the cells with PI/RNase Staining Buffer (BD Biosciences) with 10,000−30,000 events for each sample. The data were processed and interpreted using FlowJo software (BD Biosciences).

### 2.24 Integrin Array

The surface integrin subunits on BG01‐hESCs were determined using the α/β Integrin‐Mediated Cell Adhesion Array Combo Kit (ECM532, Millipore) according to the manufacturer's guidelines. Briefly, hESCs were harvested using TrypLE Select Enzyme, pelleted and 100  µL of resuspended cells (5 × 10^4^ cells) were transferred to wells containing immobilized antibodies specific to individual integrin subunits or heterodimers. Goat anti‐mouse antibody‐coated wells were the negative control. Integrin expression was quantified using the manufacturer's instructions.

### 2.25 Human Phospho‐Kinase Array

We used the Proteome Profiler Human Phospho‐Kinase Array Kit (ARY003C, R&D Systems) to measure the signalling pathways activated by MFGE‐8. hESCs were seeded at a density of 1 × 10^5^ cells/well in Matrigel‐coated 6‐well tissue culture plates containing 2 mL of mTeSR1 medium. On the fourth day, the cells were washed twice with sterile PBS and then resuspended in mTeSR1 medium supplemented with 5 µg/mL of anti‐M8nAb (MFG‐06 [sc‐8029], Santa Cruz Biotechnology) or sterile PBS for 2 h at 37°C. Cells were lysed in 100 µL of lysis buffer and prepared for phosphorylation analysis in accordance with the manufacturer's instructions.

### 2.26 siRNA Transfection

hESCs were transfected with siRNA as described previously (Belair et al. 2019). For the siRNA/lipid mixture, 20 pmol of siRNAs and 5 µL of Lipofectamine RNAiMAX Transfection Reagent (Invitrogen) were separately diluted in 50 µL of OPTI‐MEMI (Invitrogen) and incubated at RT for 5 min, then mixed gently by pipetting and incubated at RT for an additional 30 min. hESCs (at 70%–80% confluency) in mTeSR1 medium were incubated with 10 µM ROCK inhibitor Y‐27632 for 1 h, the cells were dissociated, and 4.5 × 10^5^ hESCs were pelleted at 300 × *g* for 5 min. The cells were resuspended in 100 µL of the siRNA/lipid mixture, left at RT for 15 min, and then 1.5 mL of prewarmed mTeSR1 medium containing 10 µM ROCK inhibitor Y‐27632 was added. Then, 500 µL of the mixture was incubated overnight at 37°C in a single well of a Matrigel‐coated 24‐well tissue culture plate, and the culture medium was replaced the next day. At 72 h after transfection, we measured gene expression or EV uptake. For EV uptake, the cells were exposed to 30 µg/mL of PKH‐EVs for 6 h. Cells were treated with *DNM1* siRNA (siDNM1), *DNM2* siRNA (siDNM2) or control siRNA‐A (Santa Cruz Biotechnology). The sense strand sequences for siDNM1 and siDNM2 were:

siDNM1 #1 (Ambion Silencer Select, Thermo Fisher): 5′‐GCAGUUCGCCGUAGACUUU‐3′.

siDNM1 #2 (Ambion Silencer Select, Thermo Fisher): 5′‐GCUAUGCUAUCAAGAAUAU‐3′.

siDNM2 #1: 5′‐CCGAAUCAAUCGCAUCUUCUU‐3′ (Reis et al. 2015).

siDNM2 #2: 5′‐GACAUGAUCCUGCAGUUCAUU‐3′ (Reis et al. 2015).

### 2.27 Glutathione S‐Transferase Activity Assay

We used the glutathione S‐transferase (GST) assay kit (CS0410, Sigma‐Aldrich) to measure the enzymatic activities of GSTs in EVs and SFs. Briefly, EVs were lysed in RIPA buffer, and 10 µg of EVs or SFs were assayed in a 96‐well microplate (Corning). GST activity was measured by the conjugation of GSH with CDNB (S‐2,4‐dinitrophenyl glutathione) as the increase in absorbance at 340 nm at 25°C versus time. GST activity was calculated following the manufacturer's instructions.

### 2.28 GSH/GSSG Assay

The ratio of reduced glutathione (GSH) to oxidized glutathione (GSSG) in hESCs was determined using the GSH/GSSG‐Glo Assay (V6611, Promega). hESCs were seeded at a density of 1 × 10^4^ cells per well in a Matrigel‐coated 96‐well, nunclon delta‐treated, flat‐bottom microplate (Thermo Fisher) containing 200 µL of mTeSR1 medium. After 2 days, the cells were exposed to either EVs or buthionine sulfoximine (BSO) at the specified concentration for 4 h before performing the GSH/GSSG‐Glo assay.

### 2.29 Cellular Reactive Oxygen Species Assay

hESCs were seeded at a density of 1 × 10^4^ cells per well in Matrigel‐coated 96‐well tissue culture plates containing 200 µL of mTeSR1 medium. Two days after seeding, the cells were exposed to either EVs or BSO at the specified concentration for 4 h. The cells were washed twice, incubated with ROS Red Dye working solution (Cellular ROS Assay Kit [Red], ab186027, Abcam) for 1 h at 37°C and measured with a microplate reader as fluorescence excitation/emission = 520/605 nm. Data were analysed following the manufacturer's instructions and normalized to the total cell count.

### 2.30 Statistics and Reproducibility

Statistical analysis was performed using Prism 9 software (GraphPad). The data obtained from at least three independent experiments are presented as the mean ± standard deviation (S.D.). To determine significance, we used unpaired, two‐tailed Student's *t*‐tests to compare means between two groups. For comparisons of means from multiple individual groups testing linear responses to increasing treatment dosages or durations, a one‐way analysis of variance (ANOVA) was applied without correction for multiple comparisons. Significance was attributed to values of *p* < 0.05. Sample sizes were not predetermined using specific statistical methods; rather, the sample size was based on the initial data collected. No samples were excluded from the analysis. The experiments were not randomized. The investigators were not blinded during the experiments, during data collection or in the analysis of the data and the outcomes.

## 3 Results

### 3.1 hESC‐EVs and hESC‐SFs Independently and Cooperatively Regulate Stemness

To determine autocrine effects on self‐renewal and pluripotency, we cultured hESCs under serum‐ and feeder‐free conditions and treated them with the protein secretion inhibitors brefeldin A (BFA) and monensin (MON). This treatment reduced the proliferation of hESCs by 6 h (Figure 1a) and greatly increased cell death by 12 h (Figure 1b), with few viable cells remaining after 24 h (Figure S1a). GW4869, an inhibitor of neutral sphingomyelinase, which mediates biogenesis and the release of small EVs, gave similar results (Figure 1c,d). The detrimental effects of these inhibitors were partially but significantly mitigated by a conditioned medium obtained from the same hESCs, implying a contribution of intrinsic secretory molecules to the self‐renewal and survival of hPSCs (Figures 1a–d and S1a).

To characterize the role of autocrine mediators, we separated fractions of SFs and EVs from the secretome of hESCs by differential centrifugation (Figure 1e). To confirm that the EVs originated from hESCs and not from the mTeSR1 medium, particle numbers in fresh mTeSR1 medium and hESC‐CM were compared using NTA. While fresh mTeSR1 medium contained only trace amounts of particles, conditioning the medium with hESCs at 70%−80% confluency for 24 h resulted in a dramatic increase in particle numbers, approximately 32‐fold (Figure 1f). The purified EVs displayed a typical spherical shape, with diameters ranging from 20 to 700 nm (average size, 207.4 ± 9.1 nm) and a concentration of approximately 7 × 10^9^ particles/mg (Figure 1g). These hESC‐EVs expressed major EV marker proteins TSG101, Alix and CD81, while negative markers of EVs, including calnexin and GM130, were detected only in whole cell lysates (Figure 1g). To assess the roles of endogenously secreted factors in self‐renewal and pluripotency, hESCs were treated separately with hESC‐EVs and hESC‐SFs. Although we expected a constant release of EVs and SFs in hESC culture, the increase in proliferation by exogenous hESC‐EVs and hESC‐SFs was dose‐dependent (Figure 1h,j). To determine whether hESC‐EVs and hESC‐SFs influenced pluripotency, we allowed hESCs to differentiate spontaneously under feeder‐free conditions without fibroblast growth factor 2 (FGF2) and serum replacement, with or without EVs or SFs (Figure S1b). As expected, during the 5 days of differentiation, hESCs lost their pluripotency with decreased expression of pluripotency TFs, *POU5F1* and *NANOG* (Figure S1c–e). In contrast, EVs dose‐dependently restored the expression of pluripotency TFs in differentiating hESCs (Figure 1i). Interestingly, however, SFs did not significantly reverse the progression of differentiation, showing only a slight increase in *POU5F1* expression (Figure 1k), suggesting that SFs and EVs may play distinct roles in the autocrine and/or paracrine mechanisms underlying the stemness of hPSCs.

**FIGURE 2:**
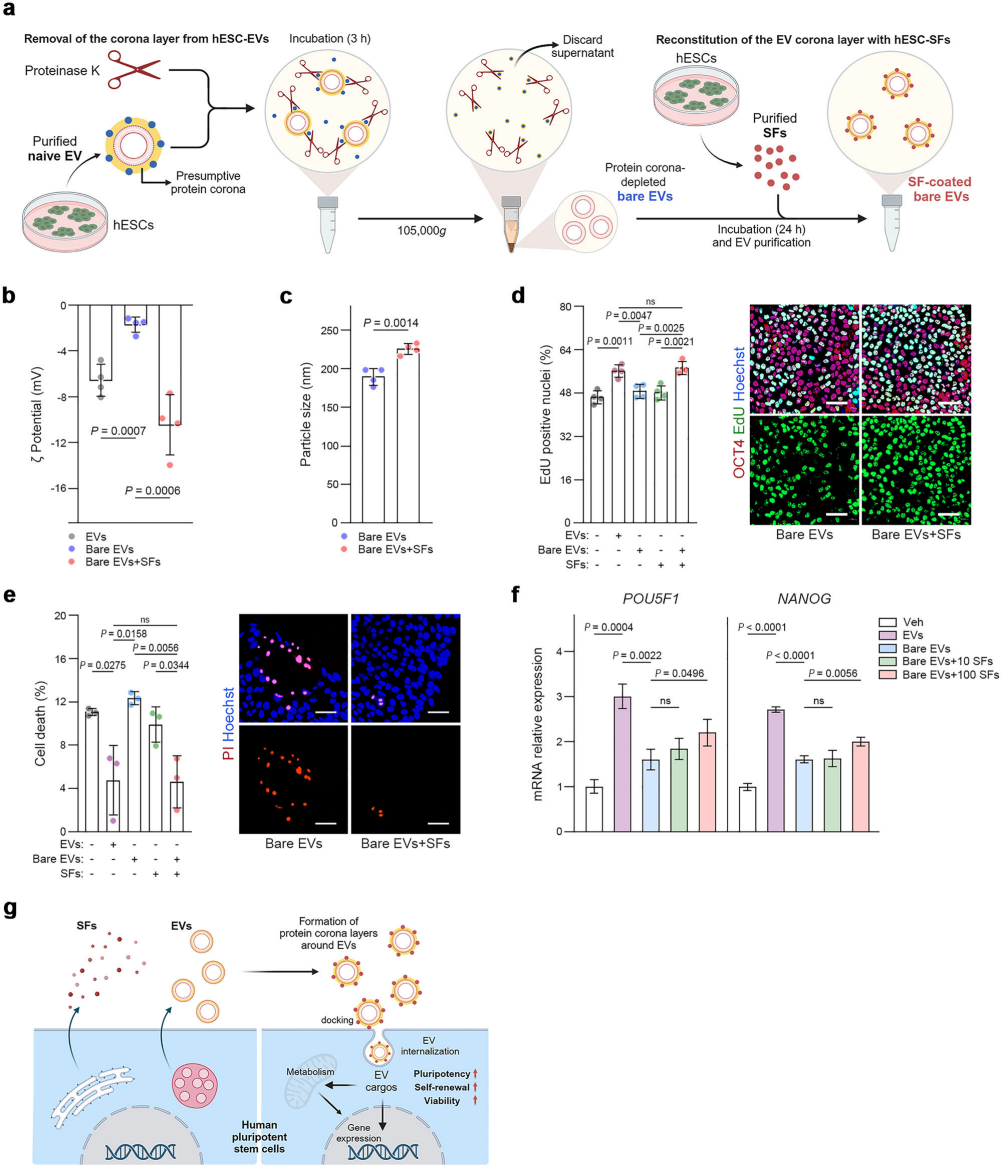
hESC‐SFs contribute to the formation of protein corona around hESC‐EVs. (a) Removal of the protein corona from hESC‐EVs (bare EVs) using proteinase K and reconstitution of the corona with hESC‐SFs (SF‐coated bare EVs). (b) Zeta potential of bare EVs and SF‐coated bare EVs (*n* = 4). (c) Measurements of sizes for bare EVs and SF‐coated bare EVs using NTA analysis (*n* = 4). (d) hESCs were exposed to EVs (50 µg/mL), bare EVs (50 µg/mL), SF‐coated bare EVs (50 µg/mL) or SFs (10 µg/mL) for 2 days, bare EVs were pre‐incubated with SFs (10 µg/mL) for 24 h to produce SF‐coated EVs. Cell proliferation was measured by EdU incorporation (*n* = 4). Cells were stained for OCT4, and nuclei were stained with Hoechst 33342. (e) Cell death was assessed by PI staining after hESCs were treated as shown in (d) (*n* = 3). (f) RT‐qPCR analysis of *POU5F1* and *NANOG* expression in differentiating hESCs after a 5‐day treatment of EVs, bare EVs and SF‐coated bare EVs (*n* = 3). Bare EVs were pre‐incubated with 10 µg/mL SFs (Bare EVs + 10 SFs) or 100 µg/mL SFs (Bare EVs + 100 SFs) for 24 h to generate SF‐coated EVs. (g) Model of protein corona formation around hESC‐EVs and its potential contribution to hPSC stemness. Graph values are mean ± S.D. with *p* values from two‐tailed *t*‐tests. ns, not significant. Scale bars, 50 µm.

The interaction between plasma proteins and blood‐circulating EVs generates a protein corona around EVs (Buzas 2022; Tóth et al. 2021; Wolf et al. 2022). As hPSCs grow in vitro as pure colonies without serum or feeder cells, we speculated that they secrete SFs that form an EV corona, affecting the delivery, targeting or uptake of EVs in vitro. To test this idea, the potential protein corona was stripped from hESC‐EVs using proteinase K (bare EVs) and re‐established by incubating the bare EVs with hESC‐SFs (SF‐coated bare EVs) (Figure 2a). The changes in zeta potential and EV sizes indicated the presence of protein corona on hESC‐EVs and confirmed the generation of the corona around bare EVs incubated with hESC‐SFs (Figure 2b,c: Bare EVs + SFs). The proliferation of hESCs increased with EV treatments, but this effect was lost after removal of the protein corona (Figure 2d). Furthermore, the loss of proliferative activity was restored by re‐establishing the EV corona with 10 µg/mL SFs from hESCs (Figure 2d). SF alone (10 µg/mL) did not increase the proliferation of hESCs (Figure 2d). Similarly, the beneficial effects of EVs on cell viability and pluripotency relied on the presence of the protein corona generated by SFs (Figure 2e,f). These data suggest that the protein corona exists on hESC‐EVs and mediates the beneficial effect of EVs on the maintenance of stemness (Figure 2g).

### 3.2 MFGE‐8 Is a Key Corona Protein of hESC‐EVs

Many secretory proteins are associated with EVs, affecting the recognition and uptake of EVs by recipient cells (Hur et al. 2021; Santucci et al. 2019; Tóth et al. 2021; Wolf et al. 2022; Berenguer et al. 2018; Lima et al. 2021). To identify the proteins that mediate EV transfer, the proteome of hESC‐EVs was analysed by LC–MS/MS analysis. Using the Chip‐Seq database (ChEA 2022, Enrichr), a close association was found between the cargo proteins in hESC‐EVs and gene regulatory networks modulating pluripotency (Figure S2a). Gene ontology enrichment analysis showed that hESC‐EVs exhibited similar features with large and small EVs reported elsewhere in terms of biological processes, cellular components and molecular functions (Figure S2b–d) (Lischnig et al. 2022; Rai et al. 2021). Additionally, hESC‐EVs were enriched with proteins related to telomere extension (Figure S2e,f), vesicle‐mediated transport, cytoskeletal organization, Rho GTPase signalling and cell division (Figure S2g,h).

**FIGURE 3:**
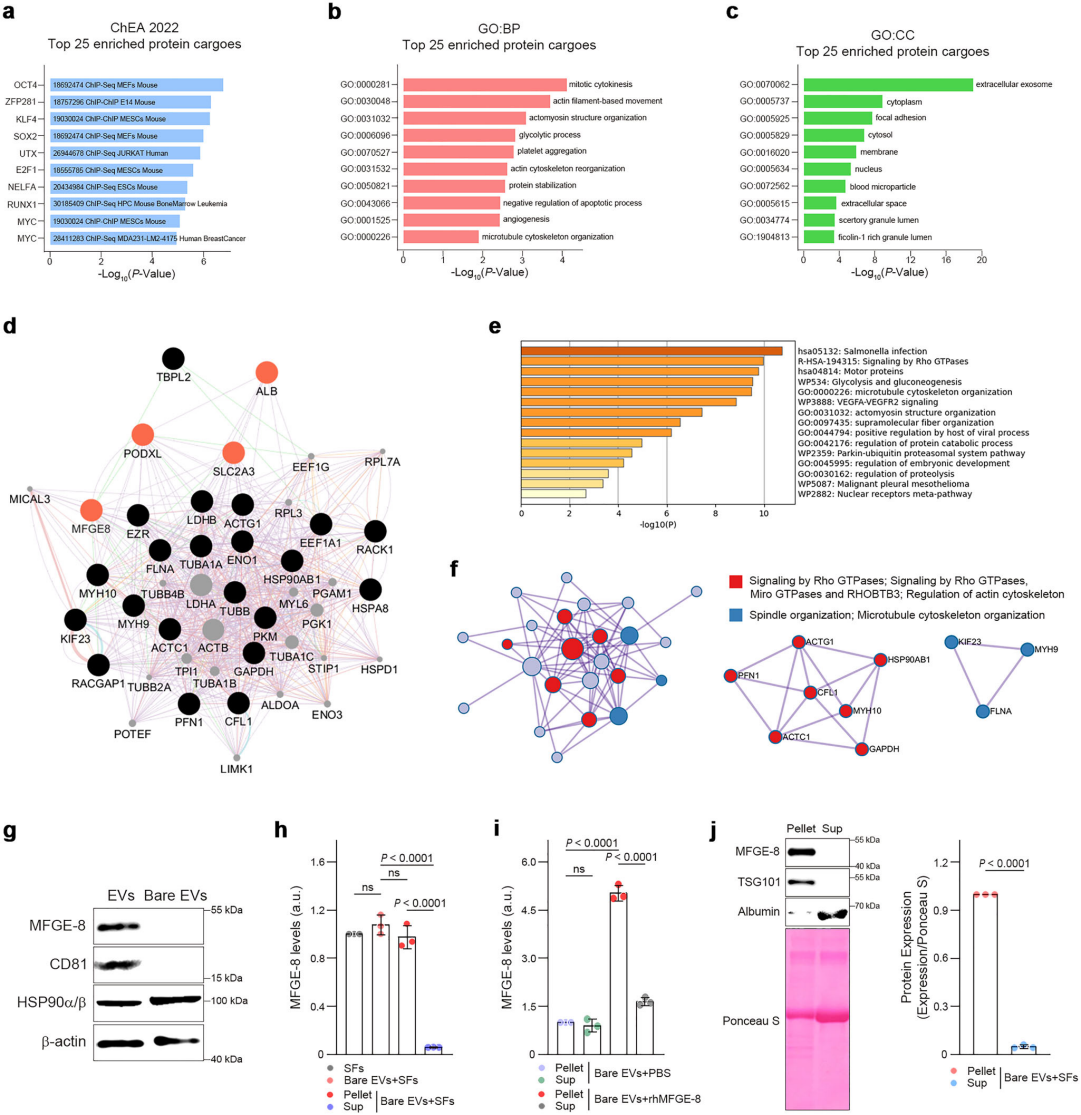
Identification of MFGE‐8 in the corona of EVs and SFs secreted from hESCs. (a) Transcription factor enrichment analysis (Enrichr, ChEA 2022) showing strong correlations between the pluripotency‐associated TFs and the top 25 enriched proteins cargos carried by hESC‐EVs. The *x*‐axis represents the significance level, and the *y*‐axis shows the enriched TFs. (b,c) Gene ontology (GO) enrichment analysis of the top 25 enriched cargo proteins in hESC‐EVs for biological process (b, BP) and cellular component (c, CC). The *x*‐axis is the significance level, and the *y*‐axis shows the enriched GO terms. (d) The protein‐protein interactions (PPIs) of the top 25 enriched protein cargoes in hESC‐EVs by GeneMania in Cytoscape. Black and orange nodes indicate the 25 identified proteins, and gray nodes are the predicted associated proteins (orange nodes indicate secretory proteins). (e) Metascape bar graph showing primary non‐repetitive enrichment clusters from the top 25 enriched protein cargoes in hESC‐EVs. The *x*‐axis and colour‐coding indicate the significance level, and the *y*‐axis shows the names of the enriched pathways. (f) Metascape visualization of the interactome network modules for the top 25 enriched protein cargoes in hESC‐EVs. The MCODE complexes were colour‐coded according to their identities, and two MCODE complexes associated with Rho GTPase and cellular organization are shown on the right. (g) Representative western blots of MFGE‐8, CD81 and HSP90α/β in hESC‐EVs before (EVs) and after (bare EVs) treatment with proteinase K. (h) ELISA of MFGE‐8 in SFs and in the mixture of bare EVs + SFs, and in pellets and supernatants (Sup) after the mixture was subjected to ultracentrifugation (*n* = 3). (i) ELISA of MFGE‐8 in pellets and Sup after ultracentrifugation of bare EVs pre‐incubated with vehicle (PBS) or 4 ng/mL rhMFGE‐8 (*n* = 3). (j) Immunoblots of MFGE‐8 in 20 µg of the pellet and Sup after ultracentrifugation of bare EVs pre‐incubated with SFs. Band intensities of MFGE‐8 were normalized to total protein content and are shown as a bar graph on the right (*n* = 3). The graph values are mean ± S.D., with *p* values from two‐tailed *t*‐tests.

It was also found that the top 25 enriched proteins were regulated by stemness‐related TFs (Figure 3a) and were closely associated with cell growth, survival, cell structural organization and vesicle‐related components (Figure 3b,c). Visualization using a protein–protein interaction (PPI) database showed close correlations between the top 25 enriched proteins, which included secretory proteins (Figure 3d: nodes in orange). Also, Rho GTPase signalling correlated highly with both the total and the top 25 enriched proteins in hESC‐EVs (Figures 3e,f and S2g). The secretory glycoprotein MFGE‐8, an opsonin molecule that bridges apoptotic cells and macrophages (Hanayama et al. 2002), attracted our attention. MFGE‐8 was previously detected on EV peripheries (Théry et al. 1999; Oshima et al. 2002; Raymond et al. 2009), suggesting its potential role in EV‐mediated cellular communications. Recently, an F‐actin regulator, Rho GTPase, was identified as a downstream signalling molecule of MFGE‐8 in vascular smooth muscle cells (Chiang et al. 2021). Interestingly, Rho GTPase plays a vital role in the long‐term survival and proliferation of hESCs by supporting the nuclear functions of YAP/TAZ through modulation of actin microfilament organization (Ohgushi et al. 2015). Both Rho GTPase signalling and actin cytoskeleton regulation were highly associated with the top 25 enriched and total protein cargoes identified in hESC‐EVs (Figures 3e,f and S2g,h).

Therefore, we focused on the role of MFGE‐8 in EV‐mediated regulation of hPSC stemness. MFGE‐8 was detected in hESC‐EVs but disappeared, along with the EV surface marker CD81, after EVs were treated with proteinase K (Figure 3g). In contrast, the luminal cargo protein HSP90α/β was still present in the proteinase K‐treated bare EVs. Comparable levels of MFGE‐8 were observed in SFs and a mixture of bare EVs plus SFs. However, MFGE‐8 was preferentially enriched in the EV pellet versus the supernatant after ultracentrifugation of the mixture (Figure 3h), indicating a strong affinity of MFGE‐8 for hESC‐EVs. These results were confirmed in a similar experiment using recombinant human MFGE‐8 protein (rhMFGE‐8) (Figure 3i), and the recruitment of MFGE‐8 to bare EVs was further validated by immunoblot analysis after ultracentrifugation of the mixture of bare EVs plus SFs (Figure 3j). These data suggest that MFGE‐8 binds to the surface of hESC‐EVs with remarkable affinity, contributing to the EV‐mediated cellular communication between hESCs.

### 3.3 MFGE‐8 Promotes EV Transfer and Is Essential for the Self‐Renewal and Survival of hPSCs

**FIGURE 4:**
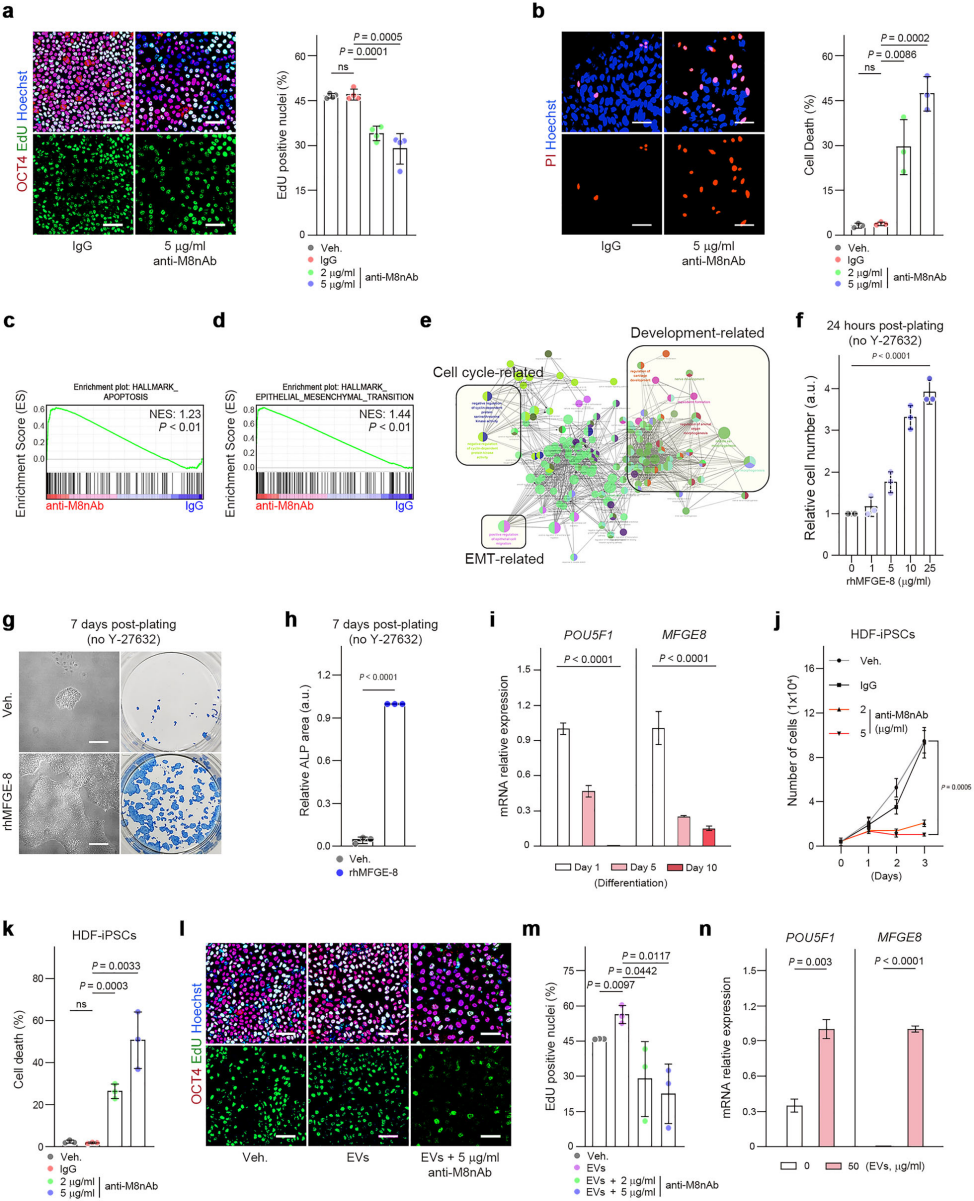
MFGE‐8 is essential for self‐renewal and survival of hPSCs. (a,b) Proliferative (a, EdU‐labelled, *n* = 4) and dead (b, PI‐labelled, *n* = 3) hESCs 2 days after treatment with vehicle (Veh), control IgG (5 µg/mL) or anti‐MFGE‐8 neutralizing antibody (anti‐M8nAb, 2 or 5 µg/mL). Cells were stained for OCT4, and nuclei were stained with Hoechst 33342. (c,d) Gene set enrichment analyses showing the enrichment of gene sets related to apoptosis and epithelial‐to‐mesenchymal transition (EMT) in transcriptome of hESCs treated with anti‐M8nAb at 24 h. (e) Connected GO term network of common upregulated DEGs was analysed by ClueGO. Each node represents a GO biological process term, with colours indicating GO groups. (f) Relative survival of single‐cell dissociated hESCs in the absence of Y‐27632 and MEF cells with increasing concentrations of rhMFGE‐8 (*n* = 3). (g,h) Feeder‐free culture of dissociated hESCs treated without (Veh) or with 10 µg/mL rhMFGE‐8 for 24 h after seeding without Y‐27632. hESC colonies were stained for alkaline phosphatase (ALP, blue) at day 7 (g). Quantitation of the ALP‐positive area is shown in h (*n* = 3). (i) RT‐qPCR analysis of *POU5F1* and *MFGE8* in hESCs that differentiated spontaneously for 5 and 10 days (*n* = 3). (j,k) Cumulative cell numbers (j) and percentage cell death (k) in human iPSC (HDF01) cultures treated with vehicle (Veh), control IgG (5 µg/mL) or anti‐M8nAb (2 or 5 µg/mL) (*n* = 3). Cell death was assessed by PI staining after 2 days of treatment. (l,m) EdU incorporation by hESCs treated with vehicle (Veh.), EVs (50 µg/mL) or anti‐M8nAb (2 or 5 µg/mL) in the presence of EVs (*n* = 3). Cells were stained for OCT4, and nuclei were stained with Hoechst 33342 (l). Quantitation is shown in (m). (n) RT‐qPCR analysis of *POU5F1* and *MFGE8* in hESCs that differentiated spontaneously for 5 days in the presence or absence of EVs (50 µg/mL) (*n* = 3). The graph values are mean ± S.D., with *p* values from two‐tailed *t*‐tests (a,b,h,j,k,m,n) and one‐way ANOVA (f,i). ns, not significant. Scale bars, 50 µm.

To determine whether MFGE‐8 is essential for maintaining hPSC cultures, hESCs grown without feeder cells were treated with an MFGE‐8‐neutralizing antibody (anti‐M8nAb). Depletion of MFGE‐8 activity using anti‐M8nAb inhibited hESC growth and led to increased cell death with prominent morphological changes within 2 days (Figures 4a,b and S3a; Videos S1−S3). RNA‐seq analysis was used to assess changes in gene expression in hESCs following 12 and 24 h of anti‐M8nAb treatment, compared to control IgG treatment. Principal component analysis (PCA) revealed that the gene expression profile of anti‐M8nAb‐treated hESCs was distinct from that of control IgG‐treated cells along the PC1 axis, which explained 71% of the variance (Figure S3b). Differentially expressed genes (DEGs) were then identified between anti‐M8nAb and IgG treatments at 12 h (265 upregulated DEGs and 337 downregulated DEGs) and at 24 h (496 upregulated DEGs and 632 downregulated DEGs). Longer exposure to anti‐M8nAb resulted in a greater number of DEGs. Hierarchical clustering of DEGs corroborated the PCA findings, indicating that anti‐M8nAb treatment led to a significantly different gene expression pattern from the control IgG at both 12 and 24 h (Figure S3c). Notably, there were 115 common upregulated DEGs (common up‐DEGs) and 179 common downregulated DEGs (common down‐DEGs) that overlapped at both time points after anti‐M8nAb treatments (Figure S3d). Gene set enrichment analysis (GSEA) using FPKM values of each group revealed that gene sets related to apoptosis and epithelial‐to‐mesenchymal transition (EMT) were predominantly enriched in anti‐M8nAb‐treated hESCs (Figures 4c,d and S3e−g). Further gene ontology (GO) enrichment analysis of common up‐DEGs demonstrated significant enrichment in GO terms related to negative regulation of cell proliferation (GO:0008285, GO:0001937, GO:0000079, GO:2000045, GO:0030308, GO:2000134) and cell migration (GO:0010595, GO:0030335) (Figure S3h). The functional GO term network created using ClueGO indicated that GO biological process terms related to negative regulation of the cell cycle, development and EMT were enriched in the anti‐M8nAb‐treated hESCs (Figure 4e). These transcriptome analysis results strongly support the observed phenotypes, including reduced proliferation, increased cell death and acquisition of a mesenchymal cell‐like morphology, induced by depleting MFGE‐8 activity in hESC culture.

Without the Rho‐associated kinase inhibitor, Y‐27632, hESCs undergo apoptosis after their dissociation into single cells (Watanabe et al. 2007). Thus, we measured the protective effect of MFGE‐8 on single‐cell dissociated hESCs without Y‐27632. The addition of rhMFGE‐8 markedly increased the survival of dissociated hESCs in a dose‐dependent manner after 1 day of cell plating (Figure 4f). By day 7, untreated dissociated hESCs produced very few small colonies, but those treated with rhMFGE‐8 during the first day after plating generated many large colonies with alkaline phosphatase activity in the absence of Y‐27632 (Figure 4g,h).

During the spontaneous differentiation of hESCs over 10 days, *MFGE8* expression decreased gradually, concomitantly with *POU5F1* (Figure 4i). To determine whether the effect of MFGE‐8 in self‐renewal and survival was specific to hPSCs, human dermal fibroblast‐derived iPSCs (HDF‐hiPSCs) and two other cell types, fibroblasts (BJ) and human bone marrow‐derived mesenchymal stem cells (BM‐hMSCs), all of which expressed *MFGE8*, were also tested (Figure S4a). Blocking MFGE‐8 activity reduced growth and caused substantial cell death in HDF‐iPSCs, similar to hESCs (Figure 4j,k); however, there were no effects in BM‐hMSCs and fibroblasts (Figure S4b,c), indicating that the effect of MFGE‐8 on growth and survival was specific to hPSCs. The specific, essential function of MFGE‐8 was confirmed by a short‐term co‐culture of hESCs and fibroblasts, in which blocking of MFGE‐8 activity gradually reduced the number of OCT4^+^ hESCs, with little or no effect on fibroblasts (Figure S4d). Importantly, anti‐M8nAb treatments inhibited the EV‐induced increase in cell proliferation and reduced the number of dividing hESCs below control levels, even in the presence of EVs (Figure 4l,m). Also, exposing differentiating hESCs to EVs increased the expression of *MFGE8* and *POU5F1* (Figure 4n).

**FIGURE 5:**
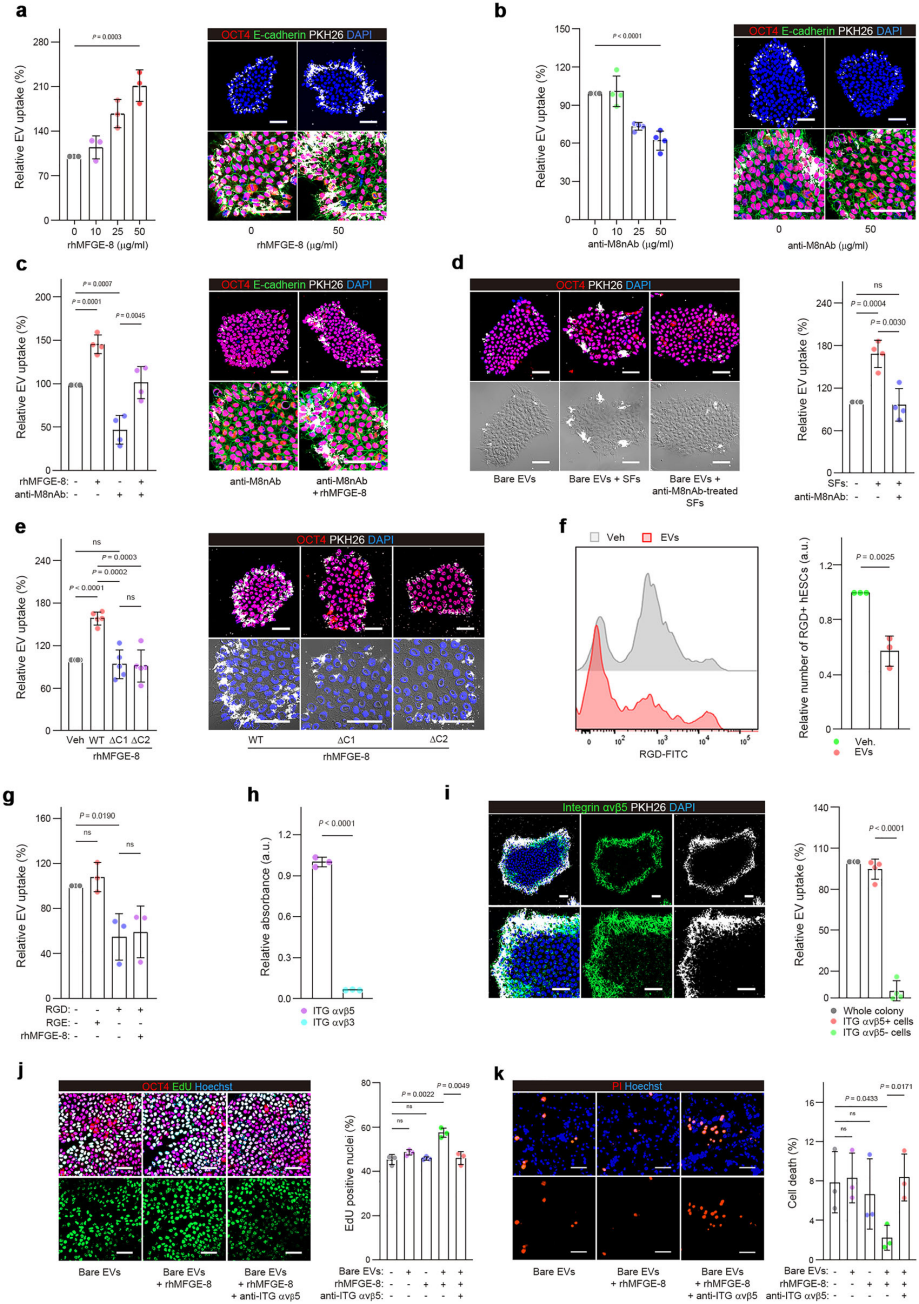
MFGE‐8 facilitates EV uptake into recipient hESCs through integrin α_v_β_5_. (a,b) The uptake of PKH‐labelled EVs (30 µg/mL) that were pre‐incubated with different concentrations of rhMFGE‐8 (0–50 µg/mL) (a, *n* = 3) or anti‐M8nAb (b, *n* = 4) for 1 h and then exposed to hESCs. The mean fluorescence intensity of hESC colonies was measured after 6 h. Cells were stained for OCT4 and E‐cadherin, and nuclei were stained with DAPI. (c) Changes in the uptake of PKH‐EVs (30 µg/mL) that were pre‐incubated with or without rhMFGE‐8 (25 µg/mL) for 1 h and exposed to hESCs for 6 h. anti‐M8nAb (5 µg/mL) was added to the hESC culture for 1 h before the cells were exposed to PKH‐EVs (*n* = 4). Cells were stained for OCT4 and E‐cadherin, and nuclei were stained with DAPI. d, hESCs were exposed to untreated PKH‐labelled bare EVs (30 µg/mL) or pre‐treated for 3 h with hESC‐derived SFs (bare EVs + SFs) or with hESC‐derived SFs in which MFGE‐8 activity was inhibited by anti‐M8nAb (50 µg/mL). The mean fluorescence intensity was measured after 6 h of exposure (*n* = 4). Cells were stained for OCT4, and nuclei were stained with DAPI. (e) hESCs were exposed to PKH‐EVs (30 µg/mL) that were pre‐incubated for 1 h with 25 µg/mL of wild‐type (WT) or variant rhMFGE‐8 lacking either the C1 or C2 domain. The mean fluorescence intensity was assessed 6 h later (*n* = 5). Cells were stained for OCT4, and nuclei were stained with DAPI. (f) Fluorescence‐activated cell sorting analysis showing the binding of 100 µM RGD‐FITC to hESCs pre‐treated for 6 h with vehicle (PBS) or 30 µg/mL EVs (*n* = 3). (g) hESCs were pre‐incubated with or without 100 µM of RGD or RGE peptides for 1 h and then exposed to PKH‐labelled EVs (30 µg/mL) for 6 h that were pre‐incubated for 1 h with or without rhMFGE‐8 (25 µg/mL) (*n* = 3). (h) A cell adhesion array showing the expression levels of integrin (ITG) α_v_β_3_ and α_v_β_5_ in hESCs (*n* = 3). (i) Images of integrin α_v_β_5_ expression and EV uptake (PKH26, 30 µg/mL, 6 h) in hESC colonies. Nuclei were stained with DAPI. Quantitation of the EV uptake in α_v_β_5_‐positive hESCs is shown as bar graphs on the right (*n* = 4). (j,k) Proliferative (EdU‐labelled) and dead (PI‐stained) hESCs 2 days after treatments with bare EVs (50 µg), rhMFGE‐8 (3 µg) or bare EVs pre‐incubated for 1 h with rhMFGE‐8. The anti‐integrin α_v_β_5_ antibody (anti‐ITG α_v_β_5_, 10 µg/mL) was added to the hESC culture 1 h before the cells were exposed to rhMFGE‐8‐treated bare EVs. Quantitative data are shown on the right (*n* = 3). Cells were stained for OCT4, and nuclei were stained with Hoechst 33342. The values in bar graphs are mean ± S.D., with *p* values from one‐way ANOVA (a,b) and two‐tailed *t*‐tests (c–k). ns, not significant. Scale bars, 50 µm.

Based on these results, we hypothesized that the endogenous secretory factor MFGE‐8 plays a critical role in EV trafficking between hESCs. To test this, hESC‐EVs were labelled with the fluorescent dye PKH26 (PKH‐EVs) and exposed to hESCs at different concentrations and time points. As expected, EV uptake increased with their dose and with the time of exposure (Figure S5a). Noticeably, EV uptake signals were preferentially detected at the margin rather than the central zones of colonies, implying positional differences in EV entry across the hESC colonies (Figure S5b). Pre‐incubating PKH‐EVs with rhMFGE‐8 increased EV uptake by hESCs and was dependent on the concentration of rhMFGE‐8 (Figure 5a). Conversely, higher doses of anti‐M8nAb decreased EV uptake (Figure 5b) and this was confirmed using two other sources of anti‐M8nAbs (Figure S5c). Even in the presence of anti‐M8nAb, 25 µg/mL of rhMFGE‐8 largely restored the uptake of PKH‐EVs (Figure 5c). In contrast, while hESC‐SFs significantly increased the uptake of bare EVs, selective inhibition of MFGE‐8 activity in the SFs blocked this increase in EV uptake (Figure 5d). The role of MFGE‐8 in EV trafficking was further validated using an additional hPSC line, HDF‐iPSCs. Similar to hESC‐EVs, HDF‐hiPSC‐EVs exhibited a typical round morphology with diameters ranging from 20 to 700 nm (average size, 206.5 ± 7.4 nm) and a concentration of approximately 4 × 10⁹ particles/mg (Figure S5d). These hiPSC‐EVs expressed key EV markers TSG101, Alix and CD81, while negative EV markers, calnexin and GM130, were detected only in whole cell lysates. As observed in hESCs, EV uptake in HDF‐hiPSCs was significantly affected by the presence or absence of MFGE‐8 activity (Figure S5e).

Human MFGE‐8 has an epidermal growth factor (EGF)‐like domain at the N‐terminus and two discoidin/Factor 5/8 type C domains (C1/C2 domains) at the C‐terminus (Hanayama et al. 2002; Oshima et al. 2002; Raymond et al. 2009). The EGF‐like domain contains an RGD motif that interacts with integrins, whereas the discoidin‐like domains bind to anionic phospholipids, such as phosphatidylserine, which exists on the outer leaflet of EVs (Nielsen et al. 2014). We hypothesized that MFGE‐8 binds to EVs via the C1 or C2 domain, guiding them to integrins on the surface of hESCs through the RGD motif. It was found that a mutant MFGE‐8 lacking either the C1 or C2 domain failed to enhance EV uptake in hESCs (Figure 5e). Furthermore, pre‐incubation of hESCs with EVs reduced the binding of FITC‐labelled synthetic RGD peptides to the cells (Figure 5f). EV uptake was significantly hindered by synthetic RGD peptides, even in the presence of rhMFGE‐8, but was not affected by RGE peptides (Figure 5g). MFGE‐8 binds mainly to integrins α_v_β_3_ or α_v_β_5_(Hanayama et al. 2002; Théry et al. 1999; Oshima et al. 2002; Raymond et al. 2009). hESCs had higher expression of α_v_β_5_ than α_v_β_3_ (Figures 5h and S5f). Interestingly, integrin α_v_β_5_ was expressed predominantly in the periphery of hESC colonies where most of the EV uptake occurred (Figure 5i). In addition, the proliferation of hESCs was significantly increased by pre‐incubating bare EVs with rhMFGE‐8, but this effect was abrogated substantially by blocking integrin α_v_β_5_ with anti‐integrin α_v_β_5_ antibody (Figure 5j). Similar effects were observed on cell survival (Figure 5k). These results indicate that MFGE‐8 serves as an indispensable bridge between EVs and hESCs, mediating the intercellular communication and supporting the capacity of hESCs for self‐renewal.

### 3.4 MFGE‐8 Promotes EV Endocytosis and Self‐Renewal of hESCs via the α_V_β_5_/AKT/GSK3β Axis

**FIGURE 6:**
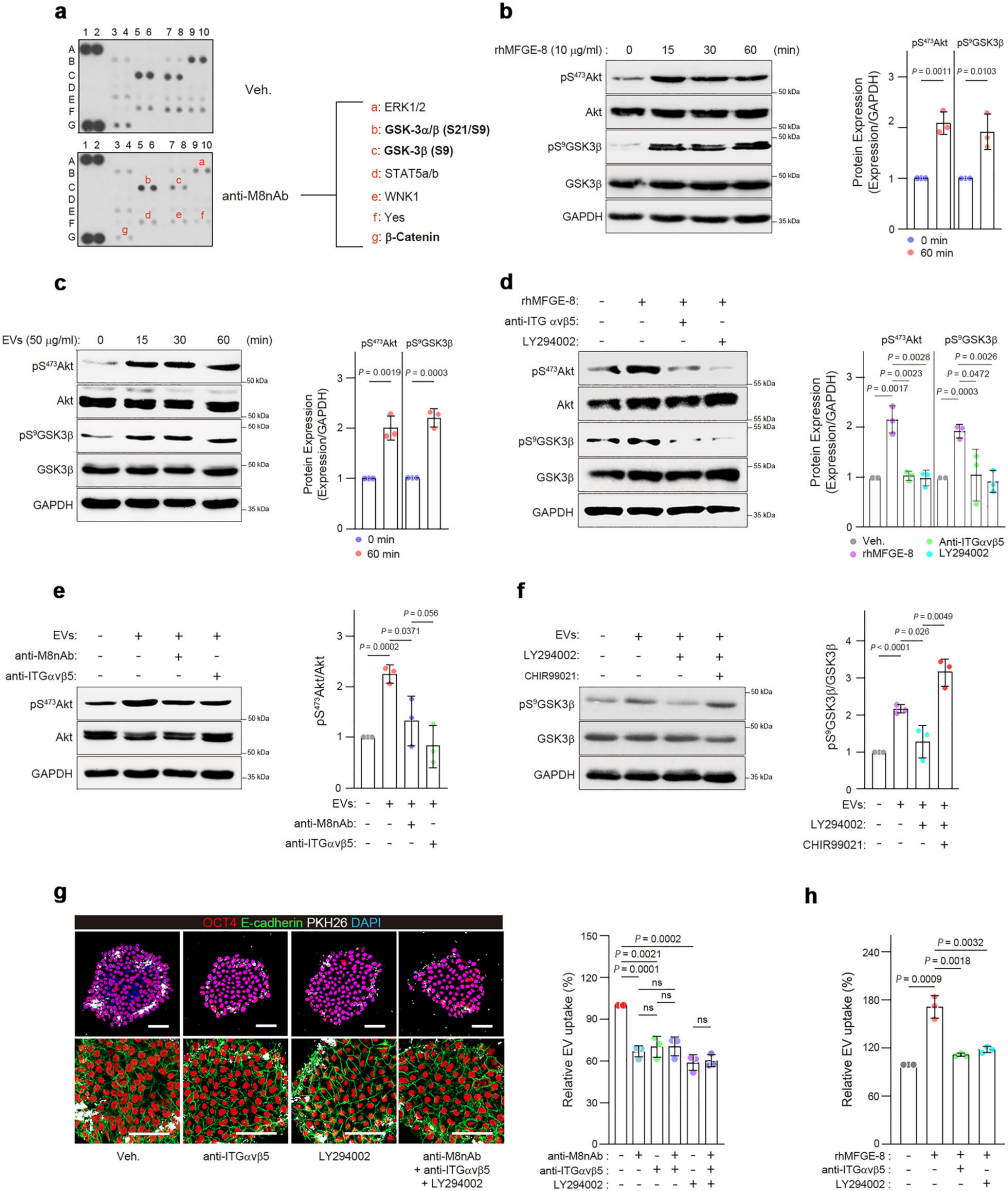
MFGE‐8 mediates EV uptake in hESCs through the integrin α_v_β_5_/Akt/GSK3β pathway. (a) A human phospho‐kinase antibody array for hESCs cultured for 2 h in the absence or presence of anti‐M8nAb (5 µg/mL) to block EV uptake. (b,c) Immunoblot showing phosphorylation of AKT and GSK3β after hESCs were treated with rhMFGE‐8 (b) or hESC‐derived EVs (c), with relative ratios of phosphorylation of AKT and GSK3β after 1 h of treatment shown on the right (*n* = 3). (d–f) Immunoblots showing the phosphorylation of AKT and GSK3β after hESCs were treated with different combinations of rhMFGE‐8 (5 µg/mL), EVs (50 µg/mL), anti‐M8nAb (5 µg/mL), anti‐integrin α_v_β_5_ antibody (anti‐ITG α_v_β_5_, 10 µg/mL), LY294002 (10 µM) and/or CHIR99021 (2 µM). hESCs were pre‐incubated with or without anti‐M8nAb, anti‐integrin α_v_β_5_ Ab, LY294002 and CHIR99021 for 1 h. Cells were treated with rhMFGE‐8 or hESC‐EVs for 15 min and 30 min, respectively. Relative phosphorylation is shown as a bar graph on the right (*n* = 3). (g) Image of PKH‐EV uptake in hESCs treated with the factors indicated. PKH‐labelled EVs (30 µg/mL) were pre‐incubated with or without anti‐M8nAb (25 µg/mL) for 1 h and exposed to hESCs for 6 h. The anti‐ITG α_v_β_5_ (10 µg/mL) or LY294002 (10 µM) was added to the hESC culture for 1 h before the cells were exposed to the PKH‐EVs. Cells were stained for OCT4 and E‐cadherin, and nuclei were stained with DAPI. Relative EV uptake is shown as a bar graph on the right (*n* = 3). (h) EV uptake in hESCs after treatment with combinations of factors as indicated. hESC‐EVs (30 µg/mL) were pre‐treated with or without rhMFGE‐8 (25 µg/mL) for 1 h before exposure to cells. The anti‐ITG α_v_β_5_ (10 µg/mL) or LY294002 (10 µM) was added to the hESC culture for 1 h before the cells were exposed to PKH‐EVs. The mean fluorescence intensity was measured after 6 h of treatment (*n* = 3). The graph values are mean ± S.D., with *p* values from two‐tailed *t*‐tests. ns, not significant. Scale bars (g), 50 µm.

We next investigated the downstream signalling cascade in hESCs after stimulation of integrin α_v_β_5_ with MFGE‐8 or EVs. Inhibition of MFGE‐8 affected the signalling molecules ERK1/2, GSK3α/β, STAT5a/b, WNK1, Yes and β‐catenin in hESCs (Figure 6a). Among these, GSK3β regulates endocytosis and the cell cycle (Reis et al. 2015; Lai et al. 2016), suggesting its potential engagement in the downstream of MFGE‐8‐induced integrin activation in hESCs. This idea was supported by RNA‐seq data showing that blocking MFGE‐8 affected the expression of genes associated with Wnt/beta‐catenin signalling and endocytosis (Figure S6a,b). GSK3β is negatively regulated by AKT via Ser9 phosphorylation (Reis et al. 2015; Lai et al. 2016). As expected, rhMFGE‐8 activated AKT within 15 min in hESCs, leading to Ser9 phosphorylation of GSK3β (Figure 6b). Similar signalling events were induced by the treatment with hESC‐EVs (Figure 6c). Blocking integrin α_v_β_5_ abolished MFGE‐8‐induced phosphorylation of AKT and GSK3β, and the AKT inhibitor LY294002 also reduced MFGE‐8‐mediated GSK3β phosphorylation (Figure 6d). Inhibiting MFGE‐8 or integrin α_v_β_5_ reduced EV‐mediated activation of AKT (Figure 6e). LY294002 inhibition of AKT reduced hESC‐EV‐induced GSK3β phosphorylation, which was restored and enhanced by the addition of CHIR99021, a selective inhibitor of GSK3β (Figure 6f). EV uptake was reduced by anti‐integrin α_v_β_5_ antibody, by LY294002 inhibition of AKT or by combined treatments with anti‐integrin α_v_β_5_ antibody and anti‐M8nAb at levels similar to anti‐M8nAb alone (Figure 6g). Inhibition of AKT also abrogated the increase in EV uptake induced by rhMFGE‐8 (Figure 6h). Thus, our results indicate that MFGE‐8‐mediated EV uptake in hESCs involves integrin α_v_β_5_/AKT/GSK3β signalling.

**FIGURE 7:**
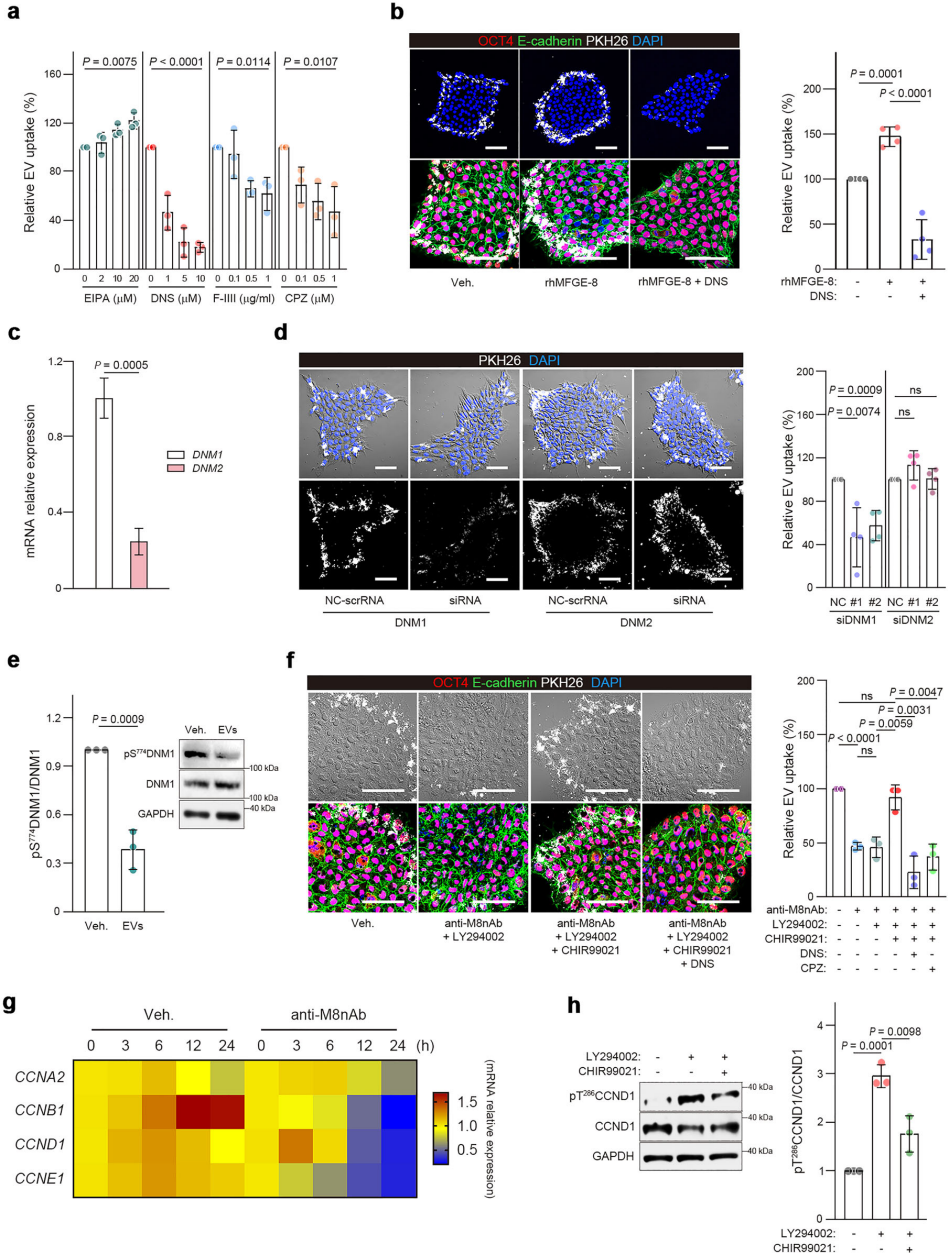
MFGE‐8 promotes endocytosis of EVs and self‐renewal of hESCs by activating dynamin‐1 and cyclin D1 via the integrin α_v_β_5_/Akt/GSK3β axis. (a) Relative EV uptake in hESC cells treated with endocytosis inhibitors for 1 h (*n* = 3). hESC cells were incubated with PKH‐EVs (30 µg/mL) for 6 h. (b) Images and quantitation of PKH‐EV uptake in hESCs. EVs were pre‐incubated with or without rhMFGE‐8 (25 µg/mL) for 1 h before they were used to treat hESCs in the presence or absence of dynasore (DNS, 10 µM) (*n* = 4). Cells were stained for OCT4 and E‐cadherin, and nuclei were stained with DAPI. (c) RT‐qPCR analysis of *DNM1* and *DNM2* expression in hESCs (*n* = 3). (d) EV uptake in hESCs after siRNA knockdown of dynamin 1 (*siDNM1*) and dynamin 2 (*siDNM2*). Cell nuclei were stained with DAPI. Two siRNAs (#1 and #2) targeting different mRNA sequences of dynamins were used (*n* = 4). (e) Immunoblots showing phosphorylation of DNM1 in hESCs treated with or without 100 µg/mL hESC‐EVs for 1 h. The relative phosphorylation of DNM1, quantitated by densitometry, is shown as a bar graph on the left (*n* = 3). (f) Images and quantitation of EV uptake for hESCs treated with combinations of anti‐M8nAb and inhibitors, as indicated. hESCs were pre‐treated with LY294002 (10 µM), CHIR99021 (2 µM), DNS (10 µM) or chlorpromazine (CPZ) (1 µM). hESC‐EVs were pre‐incubated with or without anti‐M8nAb (25 µg/mL) for 1 h before exposure to cells (*n* = 3). Cells were stained for OCT4 and E‐cadherin, and nuclei were stained with DAPI. (g) Expression of cyclins in hESCs treated with or without anti‐M8nAb (5 µg/mL) by RT‐qPCR is shown as a heatmap. (h) Immunoblots showing the relative phosphorylation of cyclin D1 (CCND1) in hESCs treated for 3 h with or without LY294002 (10 µM) or CHIR99021 (2 µM). Phosphorylation was quantitated by densitometry, shown as a bar graph on the right (*n* = 3). The graph values are mean ± S.D., with *p* values from one‐way ANOVA (a) and two‐tailed *t*‐tests (b–f,h). ns, not significant. Scale bars, 50 µm.

To characterize the endocytic machinery in hESCs that drives MFGE‐8‐mediated EV internalization, EV uptake was measured in hESCs treated with endocytosis inhibitors. Dynasore (DNS), a dynamin‐mediated endocytosis inhibitor; filipin III (F‐III), a caveolae‐dependent endocytosis inhibitor; and chlorpromazine (CPZ), an inhibitor of clathrin‐coated pit assembly, reduced EV uptake into hESCs, whereas EIPA (*N*‐ethyl‐*N*‐isopropyl amiloride), a macropinocytosis inhibitor, did not inhibit uptake (Figure 7a). The inhibitory effect of DNS was striking, suggesting that dynamin plays a key role in EV endocytosis (Figure 7a,b). As AKT activates dynamin‐1 by inhibiting GSK3β (Reis et al. 2015), we examined the potential contributions of dynamin‐1 and dynamin‐2 to EV internalization. The result revealed that both dynamin‐1 and dynamin‐2 were expressed in hESCs, but dynamin‐1 was more highly expressed (Figure 7c). Notably, knockdown of dynamin‐1 (siDNM1) in hESCs substantially reduced EV uptake, whereas knockdown of dynamin‐2 had little effect (Figures 7d and S6c). Furthermore, treatment of hESCs with EVs activated dynamin‐1 by inducing dephosphorylation at Ser774 (Figure 7e). Additionally, the GSK3β inhibitor CHIR99021 restored EV uptake even with AKT and MFGE‐8 inhibition, which was abolished substantially by DNS or CPZ (Figure 7f). Experiments in HDF‐iPSCs produced the same results (Figure S6d).

As blocking MFGE‐8 activity on EVs impaired the self‐renewal of hESCs, we sought potential downstream molecules associated with the cell cycle, MFGE‐8‐mediated EV delivery and the AKT/GSK3β pathway. Cyclin D1 is a downstream target of the AKT/GSK3β pathway (Lai et al. 2016), and GSK3β is responsible for the phosphorylation and subsequent degradation of cyclin D1 in fibroblasts and neuroblastoma cells (Guo et al. 2005; Liu et al. 2017). Blocking MFGE‐8 activity in hESCs downregulated the expression of cyclins, with significant cell death and dysregulation of the cell cycle (Figures 7g and S6e). AKT inhibition in hESCs markedly increased cyclin D1 phosphorylation at Thr286, which was significantly reduced by GSK3β inhibition (Figure 7h). These results indicate that MFGE‐8 on hESC‐EVs activates dynamin‐1 and cyclin D1 via the integrin α_v_β_5_/AKT/GSK3β axis, facilitating EV endocytosis and promoting hESC self‐renewal.

### 3.5 Luminal Cargo Proteins in hESC‐EVs

**FIGURE 8:**
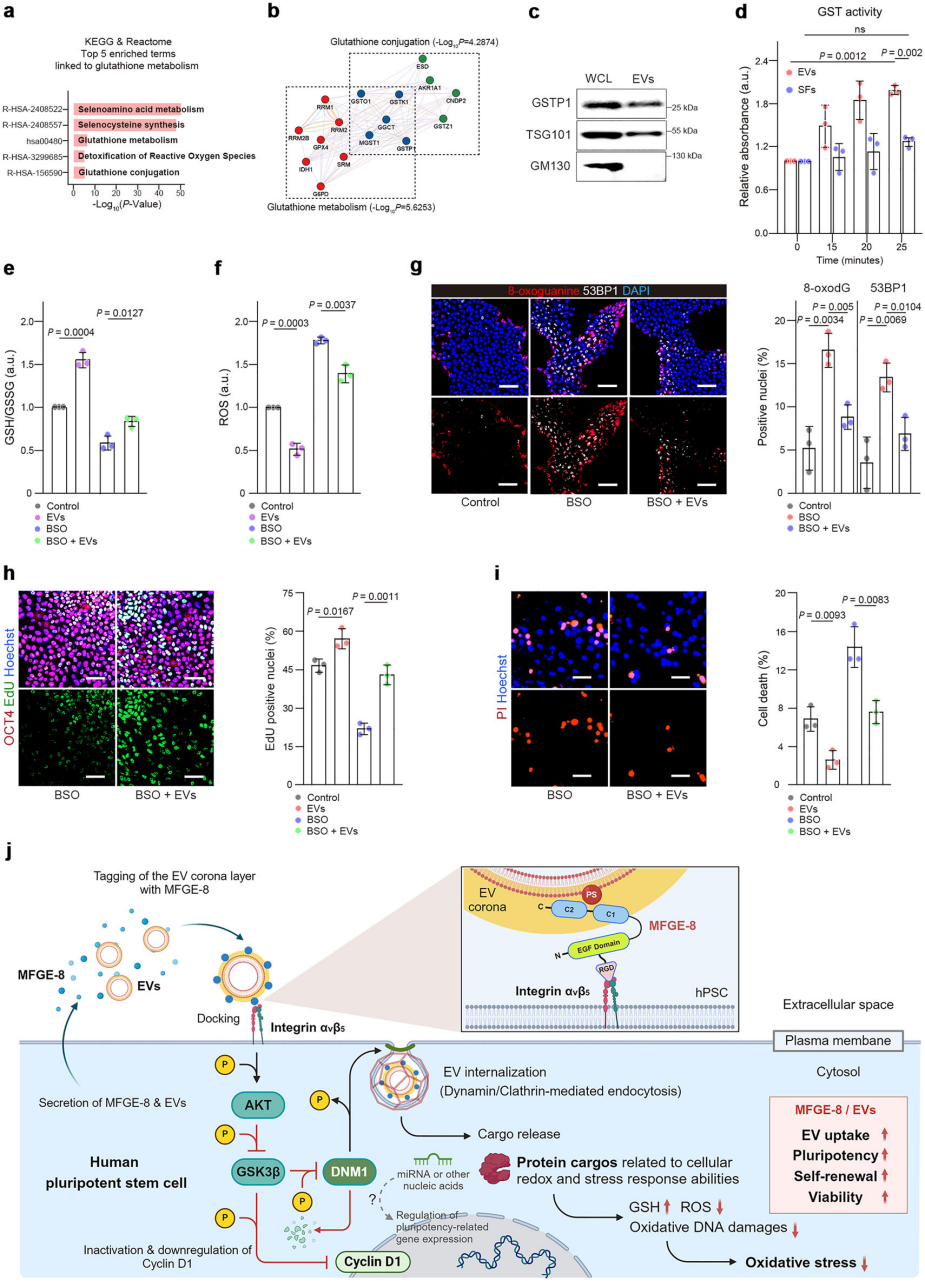
hESC‐EVs deliver cargo proteins associated with cellular redox and stress responses. (a) The top five enriched pathways associated with GSH metabolism in hESC‐EVs. Protein cargoes of hESC‐EVs were analysed using KEGG and reactome pathway analyses. *p* value < 0.05. (b) PPI networks (GeneMANIA) were constructed using the gene list extracted from KEGG and Reactome pathways related to the glutathione conjugation (a, R‐HSA‐156590) and glutathione metabolism (a, hsa00480). Red nodes indicate molecules involved exclusively in glutathione metabolism, green nodes represent molecules associated exclusively with glutathione conjugation, and blue nodes denote molecules shared by both pathways. (c) Immunoblot showing GSTP1 in hESC‐EVs. TSG101 and GM130 were positive and negative markers of EVs, respectively. (d) GST activity in hESC‐EVs (10 µg) and SFs (10 µg) was measured colorimetrically by the conjugation of the substrate CDNB with reduced glutathione (*n* = 3). (e,f) Relative GSH/GSSG (e) and ROS production (f) in hESCs treated with EVs (50 µg/mL) in the presence or absence of the GSH synthesis inhibitor BSO (40 µg/mL) for 4 h (*n* = 3). (g) Reduction of BSO‐induced 8‐oxodG and 53BP1 in hESCs by hESC‐EVs. hESCs were exposed to BSO (40 µg/mL) for 1 day in the presence or absence of hESC‐EVs (50 µg/mL). The quantitation of nuclei positive for 8‐oxodG or 53BP1 is shown in the bar graph on the right (*n* = 3). (h,i) Images and quantitation of proliferative (EdU, h) and dead (PI, i) hESCs 2 days after treatment with 50 µg/mL EVs, 40 µg/mL BSO or EVs + BSO (*n* = 3). Cells were stained for OCT4, and cell nuclei were stained with Hoechst 33342. j, Model for the role of hESC‐EVs and endogenous secretory MFGE‐8 in intercellular communication, which regulates the self‐renewal and survival of hPSCs in vitro. The graph values are mean ± S.D., with *p* values from one‐way ANOVA (d) and two‐tailed *t*‐tests (d–j). ns, not significant. Scale bars (g–i), 50 µm.

Characterizing the luminal cargoes in hPSC‐EVs is crucial for understanding their mode of action and potential clinical applications. KEGG, Reactome and PPI analyses were used to assess the contribution of cargo proteins to the growth and survival of hESCs by analyzing the proteomic signature of hESC‐EVs. The result showed that hESC‐EVs were enriched in proteins related to the antioxidant glutathione (GSH), including pathways associated with the metabolism and synthesis of selenoamino acid (an essential component of glutathione peroxidase), metabolism and conjugation of GSH, and detoxification of reactive oxygen species (ROS) (Figure 8a,b). GSEA and GO analysis also demonstrated enrichment of oxidative stress‐related gene sets and GO terms after blocking MFGE‐8 activity in hESCs (Figure S3g,h). Furthermore, hESC‐EVs carried glutathione‐*S*‐transferase (GST), a cytosolic enzyme involved in GSH conjugation for eliminating ROS‐generated toxic byproducts and exhibited its activity (Figure 8b−d). Treatment of hESCs with hESC‐EVs increased their expression of genes related to GSH metabolism, whereas anti‐M8nAb reduced these genes (Figure S7a,b). GST activity was detected primarily in EVs compared to SFs, suggesting that GSTs were delivered as cargo in EVs (Figure 8d).

Inhibition of de novo GSH synthesis by buthionine sulfoximine (BSO) resulted in a dose‐dependent increase in ROS accumulation in hESCs and reduced cell proliferation (Figure S7c,d). EV treatment increased the GSH/glutathione disulfide (GSSG) ratio in hESCs, thereby reducing ROS levels (Figure 8e,f). Furthermore, the BSO‐induced reduction of the GSH/GSSG ratio and increase in ROS levels in hESCs were reversed by EV treatment (Figure 8e,f). Additionally, the BSO‐induced GSH depletion increased 7,8‐dihydro‐8‐oxo‐2′‐deoxyguanosine (8‐oxodG), a marker of oxidative DNA damage and 53BP1, a marker of DNA damage, in the nuclei of hESCs, resulting in dose‐dependent cell death (Figure S7e,f). The BSO‐induced increases in 8‐oxodG and 53BP1 and their negative effects on proliferation and survival were all reversed by hESC‐EVs (Figure 8g–i). Thus, these results indicate that GSTs in hESC‐EVs play a critical role in the proliferation and survival of hESCs by mitigating oxidative stress.

In summary, we have shown that the endogenous secretory factor MFGE‐8 and EVs associate in the extracellular space and activate the integrin α_v_β_5_/AKT/GSK3β pathway in hPSCs (Figure 8j). This event drives EV internalization and cell cycle progression in hPSCs through dynamin‐1 and cyclin D1. Upon internalization, EVs release their cargoes into the cytoplasm of hESCs, enhancing cell growth and survival by alleviating oxidative stress. This study provides novel insights into the complex autocrine/paracrine intercellular network regulating the self‐renewal and survival of hPSCs in vitro.

## 4 Discussion

The corona proteins surrounding EVs have emerged as key mediators of autocrine and paracrine cellular communication. hPSCs sustain their stemness in vitro under feeder‐free and serum‐free conditions with limited exogenous cytokines and extracellular matrix (ECM) components. Therefore, the intrinsic autocrine interactions supporting the growth, survival and pluripotency of hPSCs have long been recognized, although the mechanisms underlying these supports are poorly understood.

In this study, we demonstrated that glycoprotein MFGE‐8 was released from hESCs and localized to the EV corona, facilitating the transfer of hESC‐EVs via the integrin α_v_β_5_/AKT/GSK3β pathway. This process allowed hESCs to internalize EVs, sustaining self‐renewal by activating dynamin‐1 and cyclin D1. Although GSK3β regulates dynamin‐1 and cyclin D1 (Reis et al. 2015; Lai et al. 2016), there are no reports of their concomitant activation by a single ligand. Here, we demonstrated that hESC‐secreted MFGE‐8 activated both dynamin‐1 and cyclin D1 by inhibiting GSK3β through the integrin α_v_β_5_/AKT axis, thereby identifying the pathway that governs the homeostasis of hPSC cultures. Cyclin D1 is essential for pluripotency and self‐renewal of hESCs, but not mouse ESCs (Ho et al. 2015; Pauklin and Vallier 2013; Stead et al. 2002). Previous studies indicated that MFGE‐8 modulates cyclin D1 expression in mouse tumor and neural stem cells via integrin signalling (Carrascosa et al. 2012, Cheyuo et al. 2015). Furthermore, the expression of cyclin D1 in endothelial cells is regulated by integrin‐induced Rho GTPase signalling (Mettouchi et al. 2001), which we identified as an enriched pathway associated with the top 25 protein cargoes in hESC‐EVs. Additionally, GSK3β modulates phosphorylation and subsequent degradation of cyclin D1 in liver tissues (Lai et al. 2016). These previous findings support our results demonstrating that the EV/MFGE‐8/integrin α_v_β_5_/AKT/GSK3β signalling axis is closely associated with EV‐mediated autocrine/paracrine pathways that regulate the self‐renewal of hPSCs in vitro.

MFGE‐8 is found in the peripheral region of EVs from multiple cell types (Théry et al. 1999; Oshima et al. 2002; Raymond et al. 2009; Morelli et al. 2004). The fact that mouse ESCs release EVs that maintain their pluripotency (Hur et al. 2021) provides a mechanistic clue for the role of MFGE‐8 in maintaining hPSC stemness. However, the presence of corona proteins and MFGE‐8 on hPSC‐EVs has not been investigated, and therefore, the potential role of MFGE‐8 in EV‐mediated stemness regulation remains unknown. Here, we showed that inhibiting MFGE‐8 activity and removing MFGE‐8 from EV surfaces reduced EV uptake by hPSCs, resulting in the loss of stemness and increased cell death. MFGE‐8 is an opsonin facilitating the phagocytic clearance of apoptotic cells in immune responses (Hanayama et al. 2002; Raymond et al. 2009). However, MFGE‐8, expressed in various cell types, also plays a role in diverse processes such as inflammation, vascularization and tumorigenesis (Li et al. 2013). We and others demonstrated that MFGE‐8 is expressed in several types of stem cells, contributing to the regeneration of damaged or diseased organs, including the liver (An et al. 2017), lung (Atabai et al. 2009) and intestine (Bu et al. 2007). MFGE‐8 also serves as a neural stem cell‐enriched niche factor that supports continuous neurogenesis in the adult brain by maintaining the stem cell pool in an autocrine manner (Zhou et al. 2018). Although the molecular mechanisms underlying these previous findings are unknown, our results strongly suggest that MFGE‐8 may serve as a critical mediator of EV trafficking in the process of tissue repair and homeostasis. Several lines of evidence showed that MFGE‐8, integrin and Rho GTPase form interconnected molecular circuits in efferocytosis and cell migration, which are essential in tissue regeneration (Chiang et al. 2021; Nakaya et al. 2006). Here, we demonstrated a strong association between the protein cargoes in hESC‐EVs and small Rho GTPase signalling. Thus, it may be worthwhile to explore the potential role of MFGE‐8 and EVs in maintaining the homeostasis of various tissue‐resident stem cells and possibly reversing multiple pathological conditions.

We found that EVs were preferentially taken up at the periphery of hESC colonies where integrin α_v_β_5_ is highly expressed. The regional difference in integrin expression and EV accumulation may be due to the distinct cell polarity and mechanical properties between the marginal area and the central zone of hESC colonies. Expanding cells at the margins of hESC colonies growing on Matrigel push out the ECM, enhancing cell‐ECM interactions at the colony edge (Rosowski et al. 2015; Toh et al. 2015). Fibronectin plays a role in EV transfer between mouse ESCs (Hur et al. 2021), suggesting that ECM components at the colony margin or in the corona of EVs contribute to EV trafficking in hPSCs. Tumour cell‐derived EVs carry integrins and ECM components, including heparan sulphate proteoglycans and fibronectin, that contribute to EV delivery (Christianson et al. 2013; Purushothaman et al. 2016). Our proteomic analysis showed that hESC‐EVs harbour various integrins and ECM components. In addition, we demonstrated that hESC‐EVs may modulate Rho GTPase signalling, which is activated by ECM components and controls the production of ECM (Kutys and Yamada 2014; Akhmetshina et al. 2008). The Rho GTPase is recently recognized as an essential signalling factor for the survival and expansion of hESCs (Ohgushi et al. 2015). Therefore, the strong interaction between hPSCs and the ECM at the colony margin may increase integrin expression, promoting EV uptake and thereby enhancing self‐renewal and resistance to cell death of marginal cells in colonies. A better understanding of the ECM composition in the hPSC‐EV corona and its role in facilitating EV uptake will help elucidate the mechanisms underlying EV‐mediated preservation of hPSC stemness. The preferential uptake of EVs at the colony margin raises the question of whether the regulation of EV production and/or secretion also depends on the location of cells in a colony of hESCs. Recent studies showed that the properties of hPSCs are affected by physical cues, including colony size and shape as well as cell topology and density (Bauwens et al. 2008; Peerani et al. 2007; Legier et al. 2023). Further studies are needed to better understand the EV‐mediated communication between EV donor and recipient cells in hPSC culture.

EVs derived from mouse ESCs may help maintain stem cell properties (Hur et al. 2021), although their cargo has not been thoroughly characterized. We found that hESC‐EVs contain many proteins associated with telomere extension and antioxidant defenses. We focused on GSTs, which are associated with the defense against oxidative stress, and found multiple enzymes that catalyse GST conjugation and metabolism in hESC‐EVs. We also confirmed that GSTs in EVs boosted GSH levels and reduced ROS production upon internalization of EVs into hESCs. Furthermore, we demonstrated that self‐renewal and survival of hESCs were impaired by blocking GSH synthesis and were rescued by EV treatments. These findings suggest that self‐renewal and survival of hESCs are regulated, at least in part, by the intercellular EV‐mediated transfer of GSTs. Interestingly, GST activity was found in small EVs from fibroblasts of young human donors and ameliorated senescence‐related tissue damage in aged cells by restoring antioxidant capacity (Fafián‐Labora et al. 2020). Similarly, EV‐mediated transfer of NAMPT enhanced NAD^+^ biosynthesis in recipient cells, delaying aging and extending the lifespan of mice (Yoshida et al. 2019). Therefore, our findings imply that hPSC‐EVs may play a role in reprogramming, rejuvenation and regeneration of injured or aged tissues. As the cargo profiles may vary depending on the size of hESC‐EVs, further fractionation and characterization of different subtypes of EVs, including microvesicles and exosomes, would better define the contributions of EV cargoes to hPSC stemness.

In this study, our primary goal was to elucidate the molecular interplay of EVs and secretory factors in hPSC culture through basic mechanistic studies. EVs and MFGE‐8 are parts of various components contained in the secretome released from hPSC in vitro. Accumulating evidence suggests that diverse secretory proteins form intricate molecular networks surrounding EVs, playing pivotal roles in trafficking intercellular dialogues (Buzas 2022; Tóth et al. 2021). Our data also showed that the ability of EVs to increase hPSC stemness was significantly impaired after removal of the peripheral proteins on EVs. Therefore, we cannot rule out the possibility that additional surface cargoes of hPSC‐EVs may function individually or in concert with MFGE‐8 or other factors to participate in preserving the stemness of hPSCs. Further mechanistic studies are needed to unveil the complex mechanisms modulating intercellular communication networks between hPSCs.

Our study focused on protein cargoes, but hESC‐EVs also contain miRNAs (Adamiak et al. 2018) that may regulate the expression of stemness‐related genes. Further investigations are needed to identify the possible links between EV cargoes and the core pluripotency TF networks. Identifying the molecular interactions of hPSC‐EV cargoes and their functions will lead to a better understanding of the autocrine/paracrine regulation of hPSC stemness and its potential therapeutic applications.

## AUTHOR CONTRIBUTIONS

**Youngseok Lee**: conceptualization (lead), investigation (lead), methodology (lead), validation (lead), visualization (lead), writing–original draft (lead), writing–review and editing (supporting). **Hyojin Kim**: investigation (supporting), methodology (supporting), validation (supporting). **Heeseok Yoon**: investigation (supporting), validation (supporting), visualization (equal). **Seunghyun Cho**: investigation (supporting), validation (supporting), visualization (equal). **Jeongjun Kim**: investigation (supporting), validation (supporting), visualization (equal). **Jihun Lee**: investigation (supporting), validation (supporting). **Sang‐Hun Choi**: investigation (supporting), validation (supporting). **Hyesun Cho**: investigation (supporting), validation (supporting). **Dong‐Hun Woo**: methodology (supporting). **Jung‐Hyuck Park**: methodology (supporting). **Choongseong Han**: methodology (supporting). **Jong‐Hoon Kim**: conceptualization (lead), funding acquisition (lead), project administration (lead), supervision (lead), writing–original draft (lead), writing–review and editing (lead).

## Conflicts of Interest

The authors declare no conflicts of interest.

## Supporting information



Supporting Information

Supporting Information

Supporting Information

Supporting Information

Supporting Information

Primers used in this study.

## Data Availability

The RNA‐seq data supporting this study have been deposited to the Gene Expression Omnibus (GEO) repository under the accession number GSE276921. The mass spectrometry proteomics data have been deposited to the ProteomeXchange Consortium via the PRIDE partner repository with the dataset identifier PXD056373.
