# Supplementary material for: MFGE‐8, a Corona Protein on Extracellular Vesicles, Mediates Self‐Renewal and Survival of Human Pluripotent Stem Cells
Source: J Extracell Vesicles. 2025 Mar 25;14(4):e70056. doi: 10.1002/jev2.70056 (PMC11934218; doi:10.1002/jev2.70056)
Supplement: Supplementary file 1 — Supporting Information [file JEV2-14-e70056-s006.docx]

**Figures S1-7**

**
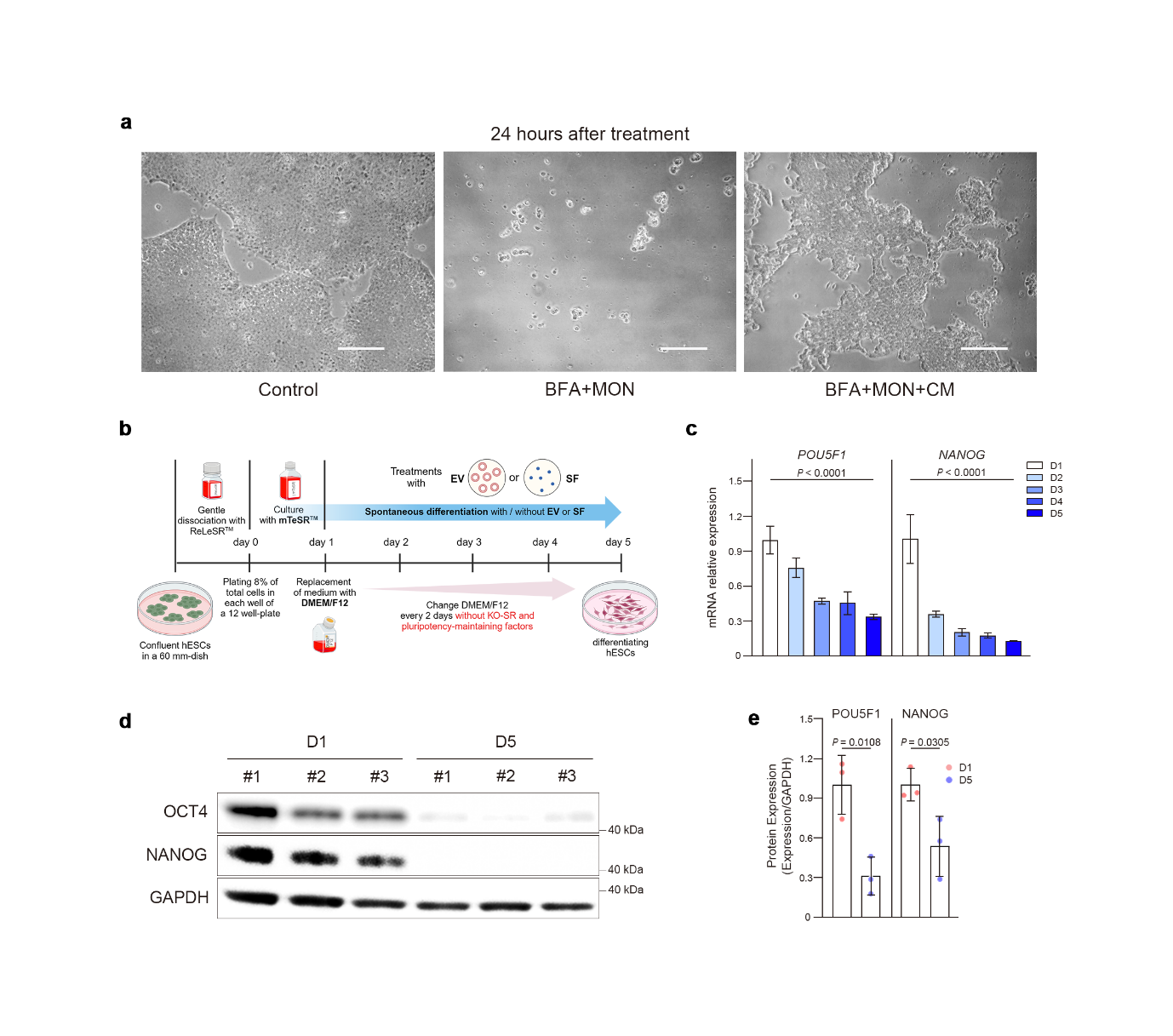
**

**Fig. S1. Endogenous secretory factors regulate survival and differentiation of hESCs. a**, Phase-contrast images of hESC colonies treated with protein secretion inhibitors BFA + MON for 24 h with or without conditioned medium (CM) from hESC cultures. **b**, Procedure for inducing spontaneous differentiation of hESCs under feeder-free conditions without fibroblast growth factor 2 (FGF2) and serum replacement (KO-SR) in the presence or absence of EVs or SFs. **c**, RT-qPCR measurements of *POU5F1* and *NANOG* expression levels in hESCs over 5 days (D1–D5) of spontaneous differentiation (*n* = 3). **d**,**e**, Immunoblots (**d**) and quantification of POU5F1 and NANOG (**e**) in hESCs on day 1 (D1) and day 5 (D5) of spontaneous differentiation (*n* = 3: #1, #2, and #3). The graph values are mean ± s.d., with *P*-values from two-tailed t-tests comparing the expression levels with those on day 1. ns, not significant. Scale bars, 50 μm.

**
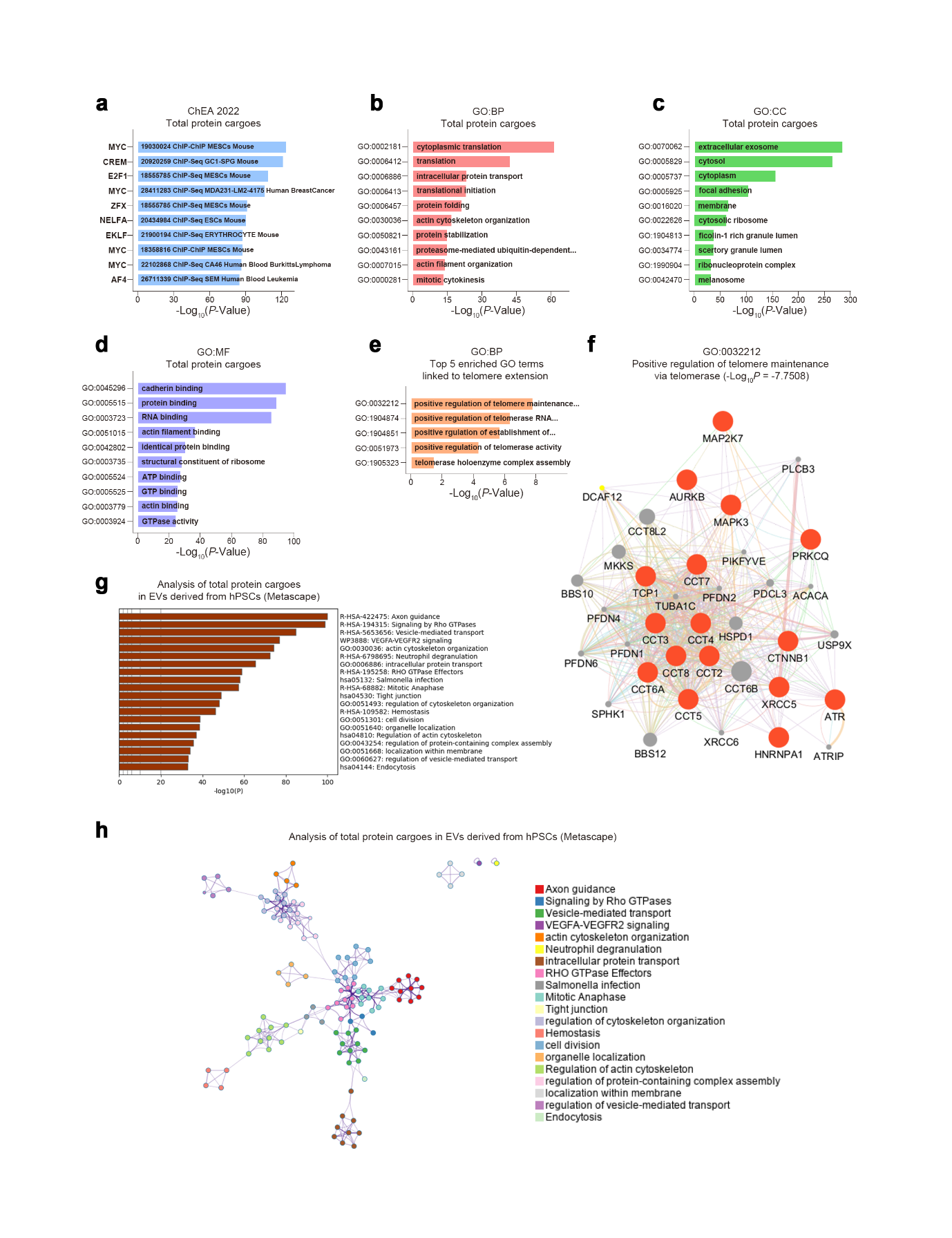
**

**Fig. S2. Bioinformatic characterization of protein cargoes delivered by hESC-EVs a**, Transcription factor enrichment analysis (Enrichr, ChEA 2022) of all proteins identified in hESC-EVs. The x-axis indicates the significance level, and the y-axis shows the enriched TFs. **b**–**d**, Gene ontology (GO) enrichment analysis of all proteins identified in hESC-EVs showed enrichment of protein cargoes linked to large and small extracellular vesicles, encompassing biological processes (BP) (**b**), cellular components (CC) (**c**), and molecular functions (MF) (**d**). The x-axis indicates the significance level, and the y-axis shows the names of enriched GO terms. **e**,**f**, The top five enriched GO terms associated with telomere extension (**e**). PPI networks of the gene list extracted from GO:0032212 were visualized using GeneMania: positive regulation of telomere maintenance via telomerase (**f**). Red nodes represent genes present in GO:0032212, and gray nodes represent genes that are predicted to associate with the 16 genes of interest (**f**). **g**, Metascape bar graph illustrating primary non-repetitive enrichment clusters of all the protein cargoes in hESC-EVs, with color-coded levels of statistical significance. The x-axis indicates the significance level, and the y-axis shows the names of the enriched pathways. **h**, Metascape visualization of clusters of enriched terms for all protein cargoes in EVs derived from hPSCs. The GO enrichment analysis was performed using a freely available web-based platform (https://david.ncifcrf.gov) designed for data analysis and visualization.


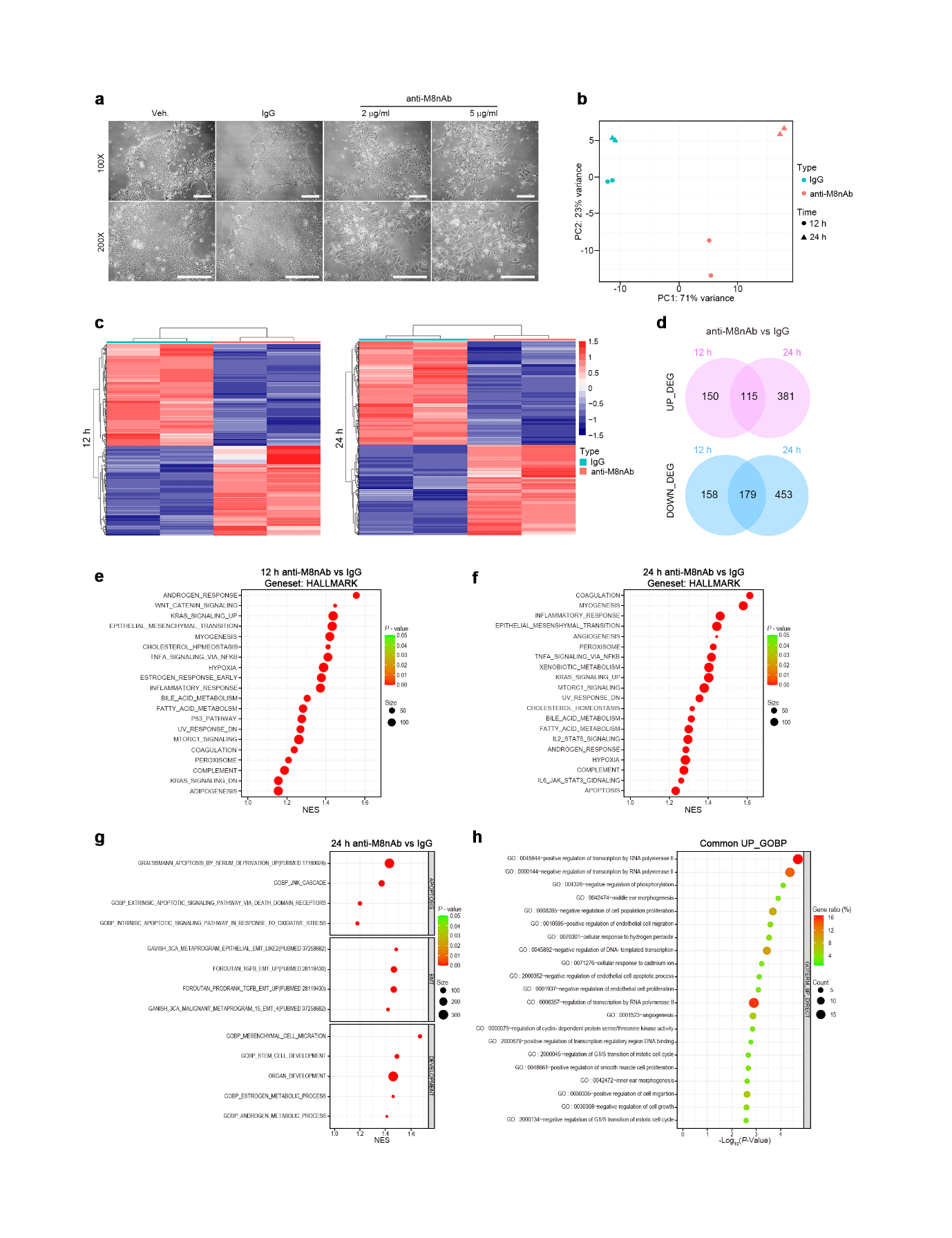


**Fig. S3. Effect of inhibiting MFGE-8 activity in hESC cultures. a**, Phase-contrast micrographs of hESCs treated with anti-M8nAb for 48 h. **b**, Principal component analysis of gene expression profiles for transcriptomes of hESCs treated with control IgG or anti-M8nAb for 12 or 24 h. **c**, Hierarchical clustering heatmap of differentially expressed genes (DEGs) in transcriptomes of hESCs treated with anti-M8nAb versus IgG at 12 and 24 h (|log_2_ fold change| ≥ 1, *q*-value < 0.05). **d**, Venn diagram illustrating the overlap of upregulated and downregulated DEGs between anti-M8nAb and control IgG treatment at 12 and 24 h. **e**,**f**,**g**, Gene set enrichment analysis (GSEA) for the transcriptomes of hESCs treated with control IgG and anti-M8nAb (NES: normalized enrichment score, *P*-value < 0.05, FDR *q*-value < 0.25). Top 20 hallmark gene sets enriched in anti-M8nAb-treated hESCs at 12 h (e) and 24 h (f) are shown as bubble plot. GSEA of anti-M8nAb versus control IgG-treated hESC transcriptomes focusing on gene sets related to apoptosis, epithelial-to-mesenchymal transition (EMT), and development. Gene sets that are significantly enriched in anti-M8nAb-treated hESCs at 24 h are presented as bubble plots (*P*-value < 0.05, FDR *q*-value < 0.25) (g). **h**, Gene ontology (GO) term enrichment analysis of common upregulated DEGs in anti-M8nAb-treated hESC transcriptome (*P*-value < 0.05). The top 21 GO biological processes enriched in common up-regulated DEGs are visualized as bubble plots (*P*-value < 0.05). Scale bars: **a**, 100 μm.


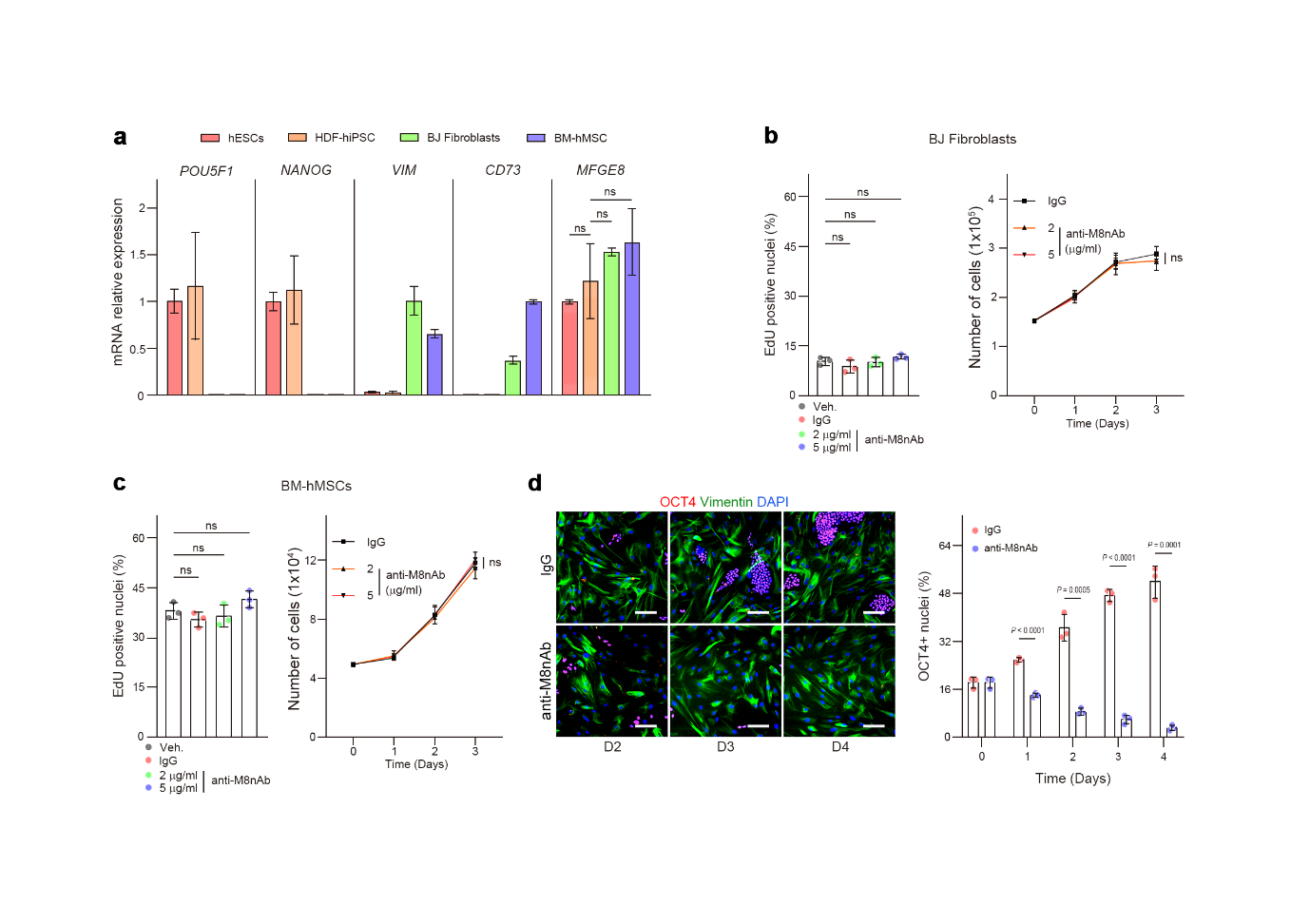


**Fig. S4. The effect of MFGE-8 in self-renewal and survival is specific to hPSCs. a**, RT-qPCR measurements of expression of *POU5F1*, *NANOG*, vimentin *(VIM*), *CD73*, *and MFGE8* in hESCs (BG01), hiPSCs (HDF01), fibroblasts (BJ), and human bone marrow-derived mesenchymal stem cells (BM-hMSCs) (*n* = 3). **b**,**c**, Effect of inhibiting MFGE-8 activity on proliferation and survival in cultures of human dermal fibroblasts and mesenchymal stem cells. Cells were treated with an isotype control IgG (5 μg/ml) or anti-M8nAb (2 or 5 μg/ml) for 48 h or indicated times, and their proliferation and survival were measured by EdU incorporation and the number of cells survived 1, 2, and 3 days after treatments (*n* = 3). **d**, Immunofluorescent staining of OCT4 and vimentin after treating co-cultured hESCs and human dermal fibroblasts with anti-M8nAb (5 μg/ml) or IgG control (5 μg/ml). The percentage of OCT4+ cells was tracked from the day of treatment until day 4 (*n* = 3). The graph values are mean ± s.d., with *P*-values from two-tailed t-tests. ns, not significant. Scale bars: **d**, 50 μm.


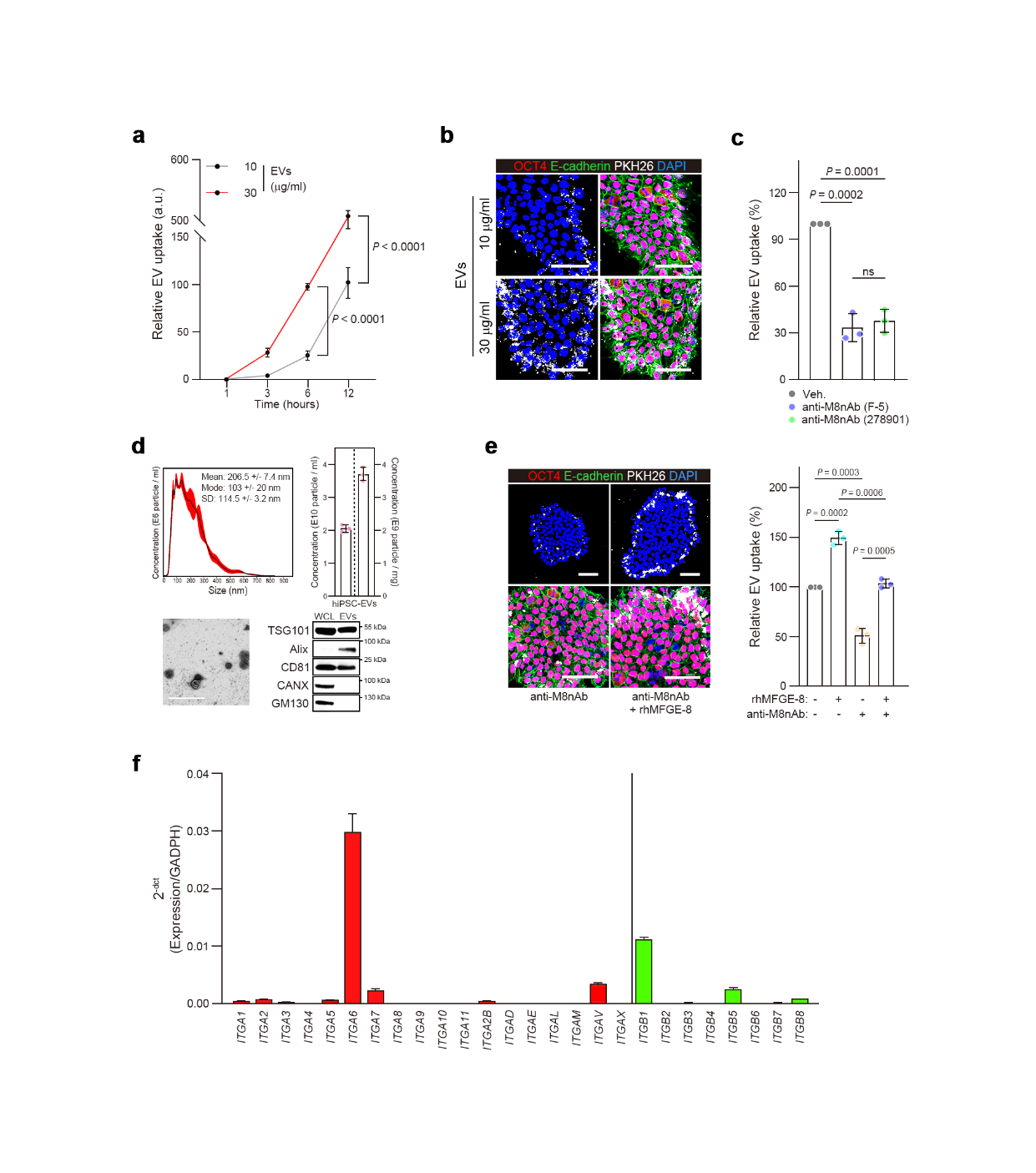


**Fig. S5. Inhibition of MFGE-8 activity perturbs EV uptake in hPSCs.** **a**, Efficiency of EV uptake in hESCs treated with 10 and 30 μg/ml of PKH-labeled hESC-EVs for 12 h (*n* = 3). **b**, Immunofluorescence images of EV uptake in hESCs treated with PKH-labeled hESC-EVs at 10 and 30 μg/ml for 6 h. Cells were stained for OCT4 and E-cadherin. Nuclei were stained with DAPI. **c**, EV uptake of hESCs in the presence or absence of two anti-M8nAbs (F-5 and 278901) at 50 μg/ml. PKH-labeled EVs (30 μg/ml) were pre-incubated with anti-M8nAb for 1 h before treatment of hESCs for 6 h (*n* = 3). **d**, Characterization of hiPSC-EVs: top left, NTA histogram showing sizes and concentrations of hiPSC-EVs; top right, Particle concentrations of the isolated hiPSC-EV samples. The particle concentration in EV solutions was determined by NTA and expressed in particle/ml and particle/mg; lower left, transmission electron micrograph of hiPSC-EVs; lower right, immunoblots of TSG101, Alix, CD81, CANX, and GM130 in whole cell lysates (WCL) and hiPSC-EVs. **e**, Inhibition of MFGE-8 activity in EV uptake using hiPSCs (HDF-iPSCs). hiPSCs were treated with PKH-labeled EVs (30 μg/ml) that were pre-incubated for 1 h with or without rhMFGE-8 (25 μg/ml) for 6 h. anti-M8nAb (5 μg/ml) was added to the hiPSC culture 1 h before the cells were exposed to PKH-EVs (*n* = 3). Cells were stained for OCT4 and E-cadherin. Cell nuclei were stained with DAPI. **f**, RT-qPCR measurements of expression of subtypes of integrins in hESCs (*n* = 3). The graph values (**a**,**c,d-f**) are mean ± s.d. *P*-values were obtained from two-tailed t-tests. ns, not significant. Scale bars (**b**,**e**), 50 μm. Scale bar (**d**), 1 μm.


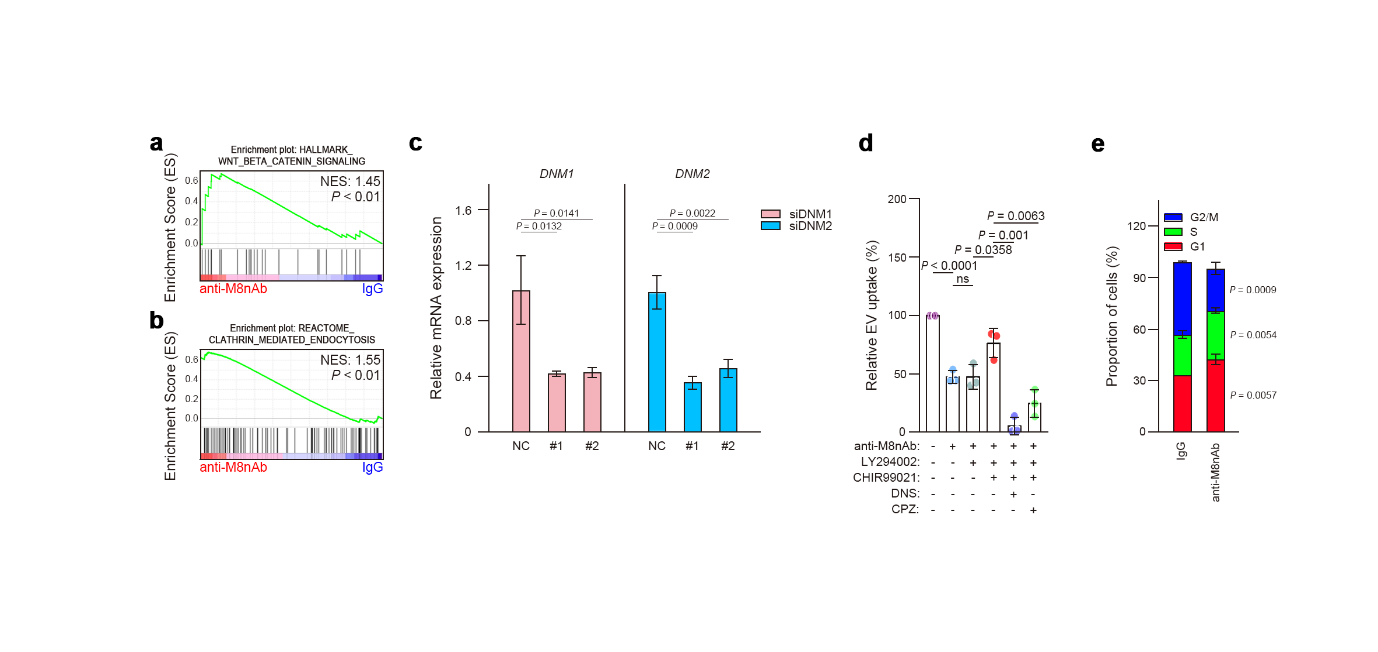


**Fig. S6. MFGE-8 regulates endocytosis of hESC-EVs and self-renewal of hESCs.** **a**,**b**, GSEA showing the enrichment of genes related to Wnt/beta-catenin signaling and clathrin-mediated endocytosis in hESCs after treatment with anti-M8nAb (*P*-value < 0.05, FDR *q*-value < 0.25). **c**, RT-qPCR verification of siRNA-mediated knockdown of dynamin-1 (*DNM1*) and dynamin-2 (*DNM2*). hESCs were transfected with siRNA #1 and #2 targeting different mRNA sequences and were analyzed 72 h after the transfections (*n* = 3). **d**, Relative EV uptake efficiency after HDF-hiPSCs were treated with the factors indicated. PKH-labeled EVs (30 μg/ml) were pre-incubated with or without anti-M8nAb (25 μg/ml) for 1 h and exposed for 6 h to hESCs that were treated with LY294002 (10 μM), CHIR99021 (2 μM), DNS (10 μM), and/or chlorpromazine (CPZ) (1 μM) (*n* = 3). **e**, Flow cytometry cell cycle profiles of hESCs treated with IgG control (5 μg/ml) or anti-M8nAb (5 μg/ml) for 48 h (*n* = 3). The graph values are mean ± s.d. *P*-values were obtained from two-tailed t-tests. ns, not significant.


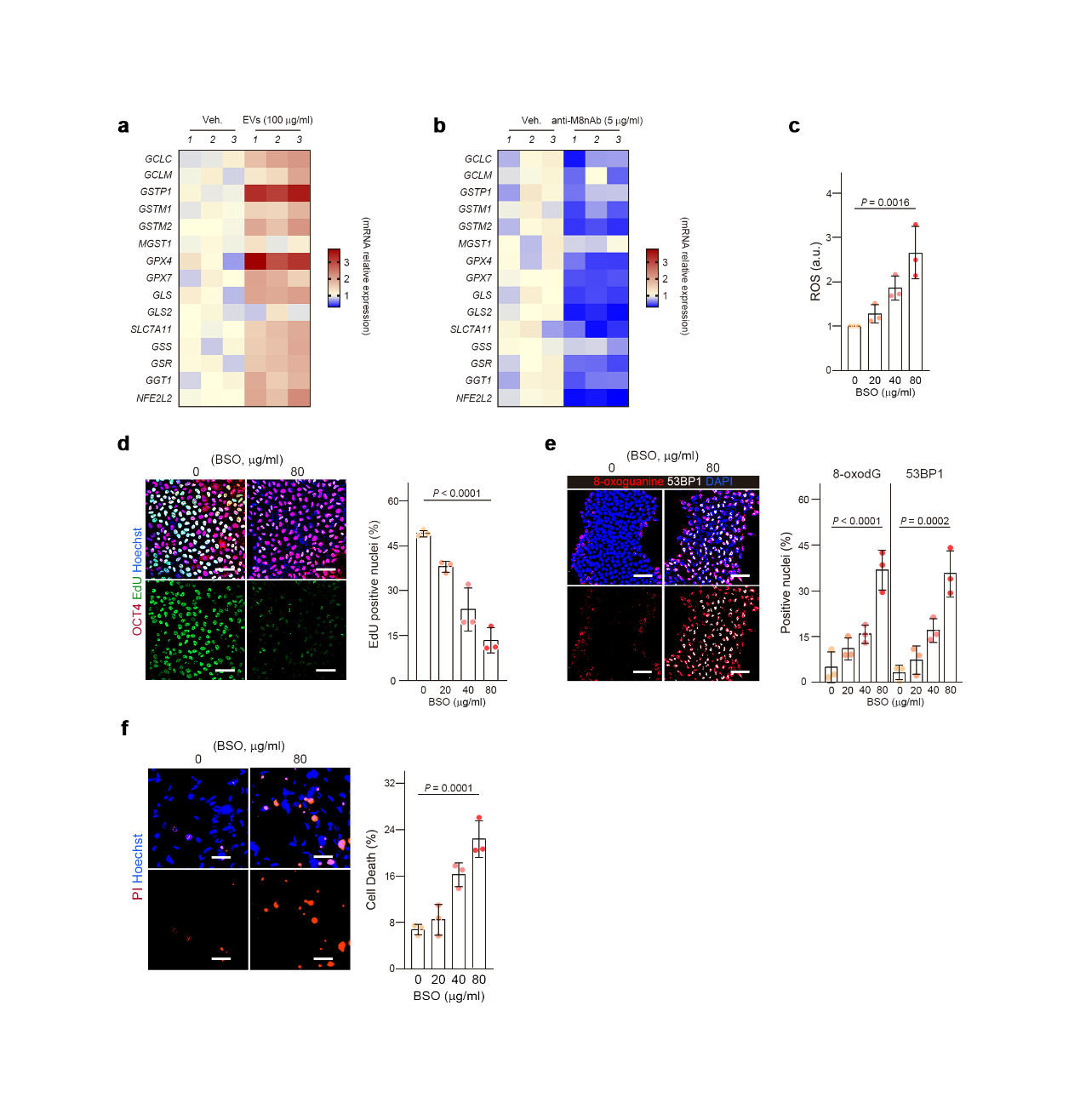


**Fig. S7. hESC-EVs mitigate the oxidative stress in hESCs and maintain cell growth and survival.** **a**,**b**, Heatmaps of RT-qPCR for changes in expression of genes related to GSH metabolism in hESCs treated with EVs (100 μg/ml) for 5 days or anti-M8nAb (5 μg/ml) for 3 days. **c**,**d**, Relative ROS production and percentages of proliferative EdU-labeled cells in hESC cultures treated for 4 h and 2 days, respectively, with various concentrations of BSO, an inhibitor of de novo GSH synthesis (*n* = 3). Cells were stained for OCT4, and nuclei were stained with Hoechst 33342. **e**,**f**, Changes in oxidative stresses (**e**) and cell death (**f**) in hESC cultures treated with various concentrations of BSO for 24 h and 48 h. Quantification of cell nuclei positive for 8-oxodG, 53BP1, and PI is shown as a bar graph on the right (*n* = 3). The graph values are mean ± s.d. *P*-values were obtained from one-way ANOVA. Scale bars, 50 μm.

**Supplementary Video 1**

Time-lapse video created using JuLi^TM^ Br. hESCs were cultured for 48 hours in mTeSR medium.

**Supplementary Video 2**

Time-lapse video created using JuLi^TM^ Br. hESCs were treated with 5 μg/ml normal mouse IgG for 48 hours in mTeSR medium.

**Supplementary Video 3**

Time-lapse video created using JuLi^TM^ Br. hESCs were treated with 5 μg/ml anti-M8nAb for 48 hours in mTeSR medium.
