# Supplementary figures and images for: MFGE‐8, a Corona Protein on Extracellular Vesicles, Mediates Self‐Renewal and Survival of Human Pluripotent Stem Cells
Source: J Extracell Vesicles. 2025 Mar 25;14(4):e70056. doi: 10.1002/jev2.70056 (PMC11934218; doi:10.1002/jev2.70056)

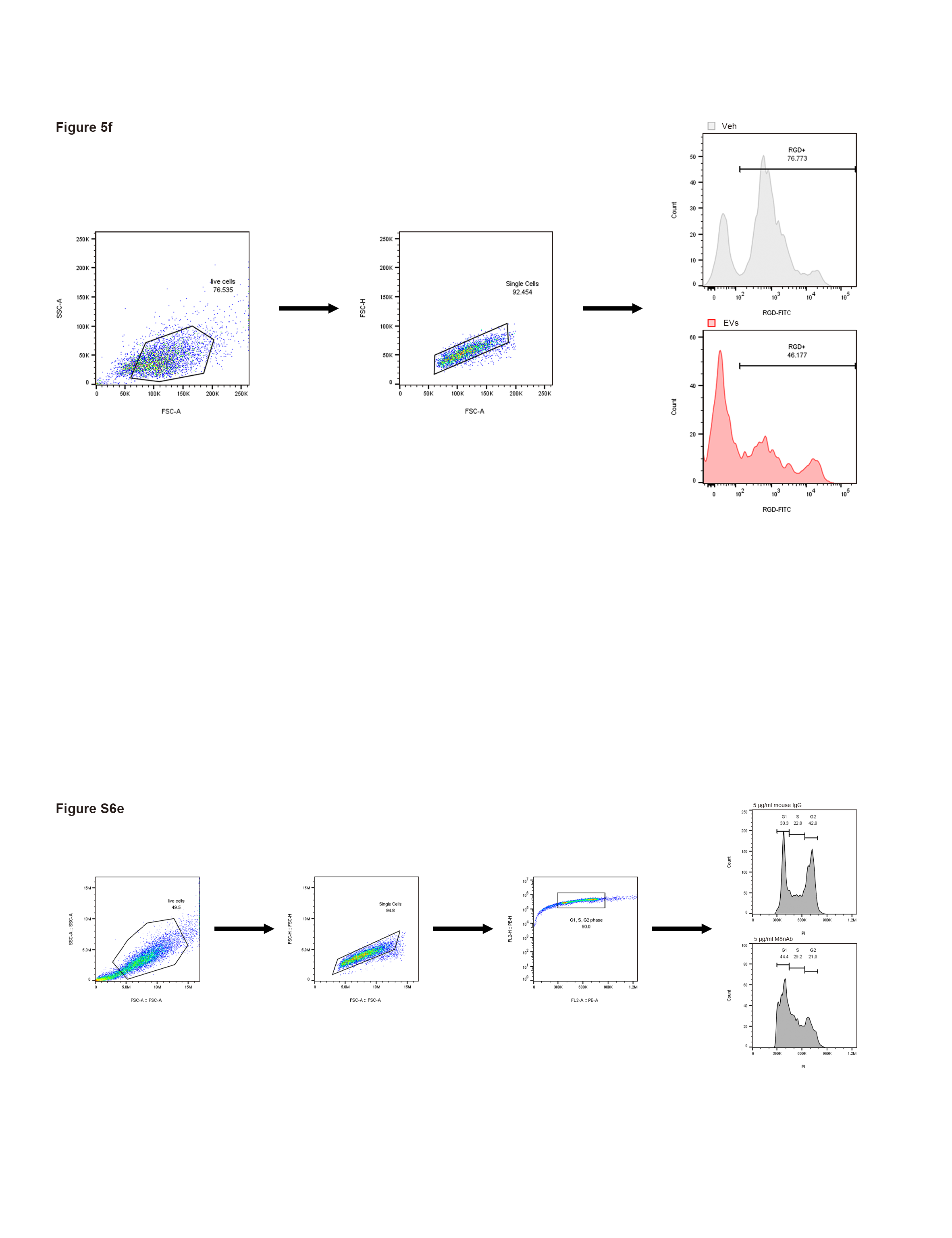

Supplement: Supplementary file 5 — Supporting Information [file JEV2-14-e70056-s002.tif]
